# Engineering of Peptide‐Inserted Base Editors with Enhanced Accuracy and Security

**DOI:** 10.1002/smll.202411583

**Published:** 2025-02-25

**Authors:** Qi Chen, Yangning Sun, Jia Yao, Yingfan Lu, Ruikang Qiu, Fuling Zhou, Zixin Deng, Yuhui Sun

**Affiliations:** ^1^ Department of Hematology Zhongnan Hospital of Wuhan University School of Pharmaceutical Sciences Wuhan University Wuhan 430071 China; ^2^ School of Pharmacy Huazhong University of Science and Technology Wuhan 430030 China; ^3^ Key Laboratory of Combinatorial Biosynthesis and Drug Discovery (Ministry of Education) Wuhan University Wuhan 430071 China

**Keywords:** base editor, CRISPR‐Cas, peptide insertion, high accuracy, low off‐targeting

## Abstract

Base editors are effective tools for introducing base conversions without double‐strand breaks, showing broad applications in biotechnological and clinical areas. However, their non‐negligible bystander mutations and off‐target effects have raised extensive safety concerns. To address these issues, a novel method is developed by inserting specific peptide fragments into the substrate binding pocket of deaminases in base editors to modify these outcomes. It is validated that the composition and position of the inserted peptide can significantly impact the performance of A3A‐based cytosine base editor and TadA‐8e‐based adenine base editor, leading to improved editing activity and precision in human HEK293T cells. Importantly, the TadA‐8e variant with DPLVLRRRQ peptide inserted behind S116 residue showed a strong motif preference of Y_4_A_5_N_6_, which can accurately edit the A_5_ base in targeted protospacer with minimized bystander and off‐target effects in DNA and RNA‐level. By summarizing the regularity during engineering, a set of systematic procedures is established, which can potentially be used to modify other types of base editors and make them more accurate and secure. In addition, the peptide insertion strategy is also proven to be compatible with traditional amino acid changes which have been reported, exhibiting excellent compatibility.

## Graphical abstract

This work developed a novel peptide insertion strategy to enhance the performance of base editors. Through changing the composition and position of the inserted peptide, peptide‐inserted base editors are engineered to exhibit robust on‐target editing activity and improved precision. Meanwhile, the harmful bystander editing and off‐target effects are significantly reduced, thereby enhancing the security and reliability of the application.

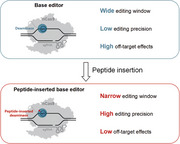

## 1 Introduction

As a revolutionary technology, base editor enables efficient base pair conversion without introducing dangerous double‐stranded DNA breaks.^[^ ^1^ ^]^ Generally, it is composed of a Cas9 nickase (nCas9) and a DNA deaminase. The former is responsible for recognizing and binding the target protospacer to form an R‐loop structure on the target strand, after which the latter can deaminate exposed bases in the non‐target single‐strand DNA (ssDNA). Specifically, cytosine base editors (CBEs) can lead to the conversion of C G‐to‐T A with the presence of a uracil glycosylase inhibitor (UGI), while adenine base editors (ABEs) can generate A T‐to‐G C conversion without inducing significant indels (insertions and deletions, typically ≤0.1%).^[^ ^2^, ^3^ ^]^

By virtue of their high efficiency and remarkable programmability, base editors have been widely employed in scientific research related to the area of animals,^[^ ^4^ ^]^ plants,^[^ ^5^ ^]^ and microorganisms,^[^ ^6^ ^]^ and have also showcased significant promise in the field of gene therapy.^[^ ^7^ ^]^ However, proliferating studies have confirmed that base editors can mediate extensive off‐target mutations at both the genome and transcriptome levels.^[^ ^8^, ^9^, ^10^, ^11^ ^]^ Specifically, CBEs generally display higher cellular toxicity and off‐target effects compared to ABEs. In addition, base editors typically exhibit an obvious editing window within the target protospacer, which frequently results in bystander editing at undesired bases, even when the primary base conversion is successfully introduced.^[^ ^12^ ^]^ These problems posed serious challenges to the safe and precise application of base editing technology, prompting the urgent need to enhance its editing accuracy and security.

To date, several strategies have been implemented to improve the performance of base editors. Shortening the flexible linker between the deaminase and nCas9 or redesigning the linker to a rigid one can moderately narrow the editing window.^[^ ^13^ ^]^ Besides, recruiting deaminases with different sequence context preferences^[^ ^14^, ^15^ ^]^ or splitting the deaminases can also enhance the accuracy of base editors.^[^ ^16^ ^]^ Recently, with the revelation of base editor's structures^[^ ^17^ ^]^ and the application of AlphaFold2,^[^ ^18^, ^19^ ^]^ researchers can clearly decipher the substrate binding pockets. It has been proven that introducing single amino acid changes within the substrate binding pockets often leads to a significant impact on the activity and accuracy of deaminases. For example, A3A‐CBE exhibited high editing efficiency at both methylated and non‐methylated target sites but was usually accompanied by severe off‐target effects and broad editing window.^[^ ^20^ ^]^ These drawbacks can be greatly improved by introducing mutations such as N57G,^[^ ^14^ ^]^ Y130F,^[^ ^20^ ^]^ and Y130A^[^ ^21^ ^]^ around the substrate binding pocket. Similarly, in the TadA‐ABE system,^[^ ^3^, ^22^ ^]^ mutations such as F148A,^[^ ^23^ ^]^ N108Q/L145T^[^ ^24^ ^]^ significantly improved the editing precision. Meanwhile, in addition to rational design, directed evolution has also been used to adjust the activity or motif preference of deaminases. For example, by employing the phage‐assisted evolution system, highly efficient deaminases such as evoCDA1 and evoAPOBEC1 have been obtained.^[^ ^25^ ^]^

Previous engineering strategies for deaminases primarily relied on introducing mutations to replace key amino acids, thereby altering the microenvironment of the substrate‐binding pocket. However, this approach remained limited in fully unlocking catalytic efficiency, likely due to insufficient modulation of conformational dynamics and cooperative residue networks. For example, a recent study engineered a small‐sized PmCDA1 cytosine deaminase with minimal off‐target effects through extensive and careful truncation.^[^ ^26^ ^]^ However, not all deaminases could be tolerant of such intense modification. Additionally, with the assistance of bioinformatics and AI, an increasing number of deaminases with varying editing behaviors have been discovered.^[^ ^19^ ^]^ If amino acid changes are employed to modify these deaminases, not only will it require a significant amount of time, but it also risks overlooking amino acids that could greatly impact the activity of the deaminases because of limited throughput. Hence, another universal method should be developed to engineer the base editors to be more powerful and precise with reduced cost of modification.

In this study, we systematically inserted specific peptide fragments into the substrate binding pocket of deaminases and thus generated several A3A‐CBE and TadA‐8e‐ABE variants with significantly higher editing activity or accuracy. We confirmed that both the composition of peptide fragments and their insertion positions within the deaminase profoundly affected the activity and functionality of the **p**eptide‐**i**nserted **c**ytosine **b**ase **e**ditors (PICBEs) and **p**eptide‐**i**nserted **a**denine **b**ase **e**ditors (PIABEs) developed in this study. Additionally, by summarizing these engineer patterns, we established a standardized process for utilizing the insertion strategy to improve base editors. Overall, we have developed a set of novel methods distinct from traditional single amino acid changes and confirmed their excellent universality, which could potentially apply to various types of base editors.

## 2 Results

### 2.1 Construction of PICBEs

The apolipoprotein mRNA editing enzyme catalytic polypeptide‐like 3 (APOBEC3; A3) proteins are a family of seven cytidine deaminases (A3A, A3B, A3C, A3D, A3F, A3G, and A3H) whose activity are associated with the restriction of viruses.^[^ ^27^ ^]^ They biochemically catalyze the deamination of deoxycytidines to deoxyuridines in ssDNA.^[^ ^28^ ^]^ Recent reports revealed their great potential in the area of base editing, coupled with Cas proteins.^[^ ^16^, ^21^, ^29^ ^]^ Among them, A3A‐CBE has been applied widely in gene disease therapy because of the high editing efficiency and insensitivity to methylation of target DNA region, whereas it showed strong bystander and off‐target effects.^[^ ^20^ ^]^ Hence, it is beneficial to improve the safety and accuracy of A3A‐CBE.

**Figure 1:**
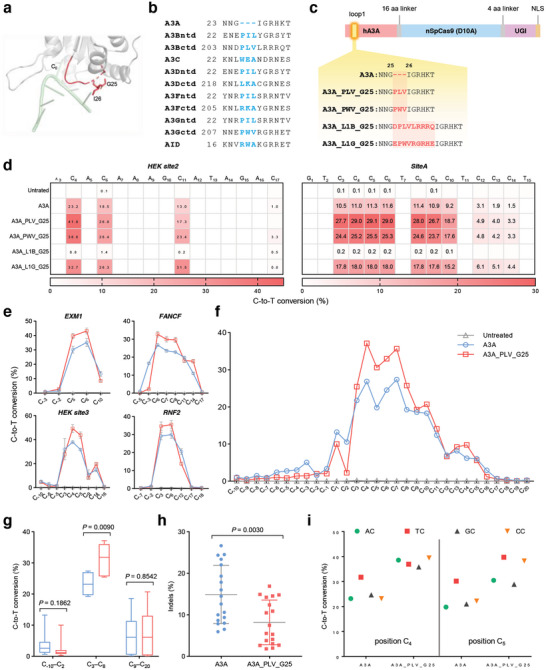
Construction of PICBEs. a) Structure of human A3A (gray, PDB code 5sww) in complex with ssDNA (green). The loop 1 is marked in red. G25 and I26 are shown as sticks. C_0_ is referred to the edited base. b) Sequence alignment of human A3 family proteins and AID. The amino acid triplet missed in A3A has been marked in red in other proteins. c) Construct of engineered A3A‐BE3. The amino acid sequences of loop 1 for A3A or PICBEs are shown in detail. The inserted sequences are bold and colored in red. d) Heatmap showing average on‐target editing frequency for A3A and PICBEs at the *HEK site2* and *SiteA*. e) On‐target editing efficiency for A3A and A3A_PLV_G25. f) Average on‐target editing efficiency for A3A and A3A_PLV_G25 at each protospacer from 18 endogenous loci shown in (d), (e), and Figure S1 (Supporting Information). g) Comparison of the editing efficiency at PAM‐distal (C_‐10_–C_2_), PAM‐proximal (C_9_–C_20_), and primary positions (C_3_–C_8_). h) Indels formation rate at 18 endogenous loci shown in (f). i) Motif preference analysis. Those protospacers containing AC, TC, GC, and CC motifs at C_4_ or C_5_ are included in statistics. All data came from three independent biological replicates. For heatmap, editing frequency higher than 0.1% were labeled in cells. For (h) and (i), bars represent mean ± s.d. *P* values are calculated using the two‐tailed Student's *t*‐test.

Sequence alignment of human A3 family proteins revealed that the loop 1 of A3A was three residues shorter than the other proteins (**Figure** **1**a,b). After inserting PWV peptide triplet from loop1 of A3B into the adjacent downstream of G25 in A3A, an *in vitro* activity assay revealed that the engineered A3A showed moderate improvement in catalytic rate and altered motif preference.^[^ ^30^ ^]^ This promising finding inspires us to apply the strategy into A3A‐BE3‐based gene editing in HEK293T, a type of human cell. From a functional consideration, we attempted to select triplet from the other well‐behaved deaminases in A3 family, and inserted the triplets into A3A. Since the N‐terminal domain of A3B (A3Bntd), A3D (A3Dntd), A3F (A3Fntd), and A3G (A3Gntd) are all inactive naturally,^[^ ^31^ ^]^ the triplets in these deaminases were not selected. Besides, A3C, A3Dctd, and A3Fctd are characterized by low activity,^[^ ^20^ ^]^ thus the triplets in these deaminases were also not selected. On the other hand, A3Bctd^[^ ^32^ ^]^ and A3Gctd^[^ ^15^ ^]^ have been developed as base editors with higher editing precision than A3A‐CBE, and they also show sufficient activity. Therefore, the triplets from A3Bctd (PLV peptide) and A3Gctd (PWV peptide) were selected finally (Figure 1c). Then, we guessed that varying the sequence with different lengths and compositions of peptides inserted into loop 1 could further modulate the editing outcomes of A3A‐CBE. Hence, longer peptides were also selected from the loop1 of A3Bctd (DPLVLRRRQ) and A3Gctd (EPWVRGRHE). All base editors were constructed based on BE3 and the name of deaminase represented the overall architecture of base editors for ease of following description. Two endogenous loci *HEK site2* and *SiteA* were tested and the editing efficiencies were investigated using high‐throughput sequencing.

Heatmaps showed that A3A_L1B_G25 produced unobservable editing at tested loci (Figure 1d). By contrast, A3A_L1G_G25 exhibited a moderately higher editing efficiency at positions C_3_–C_10_ and significantly higher editing efficiency at the protospacer adjacent motif (PAM)‐proximal positions. For example, A3A exhibited 13.0% C‐to‐T conversion at the C_11_ position of *HEK site2*, whereas A3A_L1B_G25 showed 31.5% conversion. Similar results were also observed in the comparison of A3A_PLV/PWV_G25, proving that the peptide insertion strategy changed the editing efficiency and editing window of A3A‐CBE. Moreover, divergent compositions and lengths of inserted peptides resulted in different impact on A3A (Figure 1d).

Among the four PICBE variants, A3A_PLV_G25 showed the highest C‐to‐T conversion at C_3_–C_10_ and the lowest editing efficiency at PAM‐proximal positions (Figure 1d). To unbiasedly characterize the effects of the insertion of PLV triplet on A3A, we examined their editing behavior at an additional 16 endogenous target sites (Figure 1e; Figure S1, Supporting Information). Besides the protospacer sequences, we also noted obvious C‐to‐T editing in the upstream 10 nucleotides. Therefore, the C‐to‐T conversions from position C_‐10_ to C_20_ were included in the investigation (Figure 1f). Comprehensive analysis showed that at positions C_−10_–C_2_, A3A_PLV_G25 showed significantly lower editing efficiency compared to A3A, while at C_3_–C_8_, the former's activity was 34.4% higher than latter (Figure 1g).

Additionally, it has been reported that CBEs could induce significant NHEJ‐mediated indels.^[^ ^5^ ^]^ After analyzing all 18 target sites, we found that the indels formation rate of A3A_PLV_G25 (8.2%) was nearly a half of A3A (14.9%), suggesting that the PLV triplet insertion could reduce genotoxicity of A3A‐CBE and hence improved security in the potential application (Figure 1h). Moreover, further analysis revealed that at the C_4_ position, A3A preferred the TC_4_ motif, while A3A_PLV_G25 shown almost equivalent activity for NC_4_ (N = A/C/G/T) motifs (Figure 1i), suggesting that A3A_PLV_G25 has broader activity across different motifs. At the C_5_ position, A3A also preferred TC_5_ motif, while A3A_PLV_G25 just exhibited higher activity for YC_5_ (Y = C/T) motifs compared to RC_5_ (R = A/G) motifs. In summary, A3A displayed a motif preference of TC>AC≈CC≈GC, while A3A_PLV_G25 considerably shifted to TC≈CC>AC≈GC, implying that the introduction of PLV triplet alleviated the stringent motif preference of A3A to some extent, consequently enhancing the activity of A3A_L1B_G25 at a broader range of target sites with diverse motifs.

In conclusion, A3A_PLV_G25 showed a higher activity with reduced DNA toxicity, a more focused editing window, and more diverse motif preferences. However, at the PAM‐proximal positions, there was no significant difference between A3A and A3A_L1B_G25 (Figure 1f,g), which prompted us to further modify A3A_PLV_G25 to improve its editing accuracy.

### 2.2 Improvement of PICBEs through Amino Acid Changes

**Figure 2:**
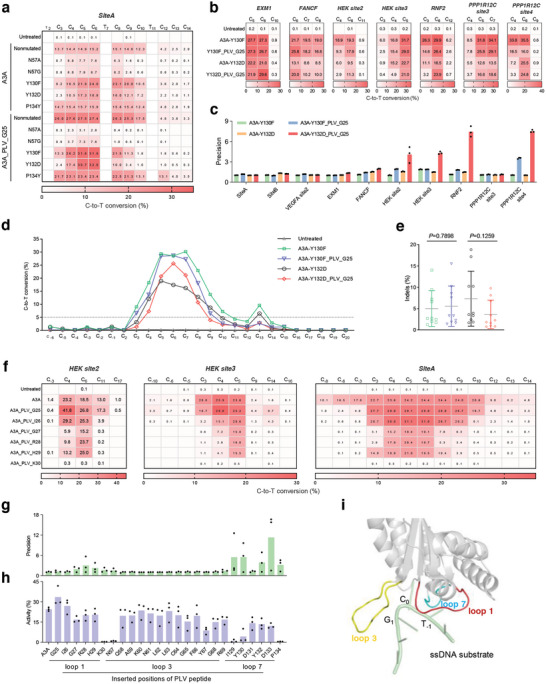
Improvement of PICBEs. a) C‐to‐T conversions for point mutated A3A and A3A_PLV_G25 at *SiteA* with multiple cytosines. b) C‐to‐T conversions for A3A and A3A_PLV_G25 with Y130F or Y132D mutation at seven endogenous loci. c) Editing precision of A3A with single amino acid changes and PLV peptide inserted. d, e) Average C‐to‐T conversions (d) and indels formation rate (e) for A3A and A3A_PLV_G25 with Y130F or Y132D mutation at ten endogenous loci shown in (a), (b), and Figure S2 (Supporting Information). f) C‐to‐T conversions for A3A and loop 1‐based PICBEs at three genomic loci. g) Editing precision comparison for A3A and PICBEs at *HEK site2*, *HEK site3*, and *SiteA*_._ h) Editing activity comparison for A3A and PICBEs at *HEK site2*‐C_6_, *HEK site3*‐C_5_, and *SiteA*‐C_5._ i) Schematic representation of A3A structure (light gray) with ssDNA (green). The loop 1, loop3, and loop7 are marked in red, yellow, and blue, respectively. T_‐1_, C_0_, and G_‐1_ bases of ssDNA substrate are indicated. The All data came from three independent biological replicates. For heatmap, editing frequency higher than 0.1% are labeled in cells. For (f) and (j), bars represent mean ± s.d. *P* values are calculated using the two‐tailed Student's *t*‐test.

Recent studies has shown that introducing single amino acid changes in the ssDNA binding pocket of A3A could narrow its editing window.^[^ ^14^, ^20^ ^]^ Considering that the effective single amino acid changes N57G, N57A, Y130F, and Y132D are not located in loop1 of A3A, we hypothesized that these mutations might function with A3A_PLV_G25. Subsequently, we introduced them into A3A and A3A_PLV_G25 and then tested these variants at three genomic loci rich in cytosine bases. The results indicated a significant decrease in editing efficiency for those containing N57G and N57A mutations (**Figure** **2**a). Thus, we directed our attention toward A3A variants containing Y130F and Y132D mutations.

Compared to wild‐type A3A, A3A‐Y130F maintained excellent activity within the primary positions and reduced editing efficiency at the PAM‐proximal positions. The improved performance was also evident in the comparison between A3A_PLV_G25 and A3A‐Y130F_PLV_G25 (Figure 2a). Therefore, as a result of cumulative effects, A3A‐Y130F_PLV_G25 exhibited significantly reduced editing efficiency at both the PAM‐distal and proximal positions compared to wild‐type A3A. Similar trends were observed in the variants containing the Y132D mutation. Additionally, a comparison of A3A‐Y130F_PLV_G25 and A3A‐Y132D_PLV_G25 demonstrated that the editing window of the latter was more compact than the former (Figure 2a; Figure S2, Supporting Information).

Considering the three tested cytosine‐rich target sites might not be representative, we additionally investigated seven additional targets with divergent compositions. Surprisingly, A3A‐Y132D_PLV_G25 demonstrated near single‐base editing resolution at targets such as *HEK site2*, *HEK site3*, and *RNF2* (Figure 2b). Especially, we focused our attention on the *RNF2* gene, as the E3 ubiquitin ligase it encodes plays an important role in sex differentiation^[^ ^33^ ^]^ and is involved in cancer development.^[^ ^34^ ^]^ In this study, we introduced C‐to‐T conversion in the intron sequence adjacent to RNF2 exon 2 (Figure S3, Supporting Information). The proportion of A3A‐Y132D‐edited cells with only C_6_‐to‐T_6_ conversion was 6.18%, while the edited cells with simultaneous C_3_+C_6_‐to‐T_3_+T_6_ was 7.72%. In contrast, A3A‐Y132D_PLV_G25, which was constructed by inserting PLV peptide into G25, resulting in 20.57% cells with only C_6_‐to‐T_6_ conversion, and the proportion of edited cells with simultaneous C_3_+C_6_‐to‐T_3_+T_6_ was 2.24%. These data suggest that, to generate cell colonies with only the C_6_‐to‐T_6_ mutation, using A3A‐Y132D_PLV_G25 can provide a 3.3‐fold higher likelihood compared to A3A‐Y132D. Furthermore, the unwanted indels introduced by A3A‐Y132D, which may disrupt the 5’ splice site GT sequence, were 2.2‐fold more frequent than those in A3A‐Y132D_PLV_G25, suggesting that A3A‐Y132D_PLV_G25 may hold considerable potential for genetic disease modeling or rescue.

To further clearly illustrate the enhancement of editing accuracy brought by the inserted peptide in the A3A‐Y130F/Y132D, we quantified their editing accuracy by “precision” (the editing efficiency of the most edited base/that of the second most edited base) (Figure 2c). We observed that the precision of A3A‐Y130F/Y132D was low, whereas the introduction of PLV peptide significantly improved their precision. In fact, the precision of A3A_PLV_G25 at the same target sites was only slightly higher than that of A3A (Figure 1d,c), suggesting that the substantial increase in precision observed in A3A‐Y132D_PLV_G25 was due to the synergistic effect of both the PLV insertion and the Y132D mutation.

Besides, the comprehensive analysis revealed that A3A‐Y130F_PLV_G25 and A3A‐Y132D_PLV_G25 had a significantly narrowed editing window compared to A3A‐Y130F and A3A‐Y132D, while maintaining a considerably high editing efficiency at the primary positions like C_6_ (Figure 2d). Furthermore, examination of the indels formation rate revealed that A3A‐Y132D_PLV_G25 (3.65%) exhibited significantly lower DNA toxicity compared to A3A‐Y132D (7.33%) (Figure 2e).

Overall, we confirmed that the insertion of PLV triplet could be compatible with mutation Y130F or Y132D together to narrow the editing window of A3A, resulting in engineered CBEs with high precision and reduced DNA toxicity.

### 2.3 Improvement of PICBEs Through AdeI Regulation

In addition to introducing single amino acid changes, recent research has shown that the gene VIII‐encoded protein (G8P) of filamentous bacteriophage and its periplasmic domain (G8P_PD_), encoding a peptide of ≈20 amino acids, can inhibit the activity of SpCas9, thereby narrowing the editing window of base editors.^[^ ^35^ ^]^ Likewise, it has been proven that AdeI (EBV ribonucleotide reductase BORF2) could inhibit the activity of A3Bctd‐CBE by forming a complex with potent interaction, thus significantly reducing the off‐target effects.^[^ ^36^, ^37^ ^]^ We hence hypothesized that AdeI could be further applied to narrow the editing window of base editors, similar to using G8P_PD_. However, according to mechanism studies,^[^ ^37^ ^]^ AdeI required the recognition of PLV in the loop1 of A3Bctd, not existed in A3A, thus unable to inhibit the activity of A3A‐CBE. Considering the existence of PLV triplet, we speculated that A3A_PLV_G25 can be recognized and inhibited by AdeI.

The results revealed that at the *HEK site2* locus, A3A remained unaffected by AdeI, while A3A_PLV_G25 was susceptible to AdeI in a dose‐dependent manner (Figure S4, Supporting Information). Furthermore, the extent of inhibition to A3A_PLV_G25 caused by AdeI varied between position C_4_ and C_6_, with a more pronounced inhibition observed at position C_4_, suggesting that AdeI may narrow the editing window by exerting different levels of inhibition at various positions. We further examined the inhibitory ability of AdeI with three doses at eight additional target sites (Figure S5a, Supporting Information). The results demonstrated that AdeI at 1.0 and 1.5‐fold doses resulted in a significant loss of editing efficiency of A3A_PLV_G25, while AdeI at 0.5‐fold dose maximally retained activity at the primary positions like C_5_, with substantially reduced editing efficiency at other positions (Figure S5b, Supporting Information). Remarkably, A3A_PLV_G25 with 0.5‐fold AdeI even showed no loss of activity at C_3_–C_8_ and minimized editing efficiency at C_‐10_–C_2_ compared to A3A (Figure S5c, Supporting Information).

Upon realizing the crucial role of PLV in facilitating AdeI's inhibitory function, we endeavored to explore the mechanism of AdeI. Consequently, we systematically substituted each amino acid in PLV and evaluated AdeI's inhibitory ability against these variants (Figure S5d–f, Supporting Information). Initially, replacing the first amino acid P with W in the triplet resulted in almost complete resistance to AdeI inhibition. Then, replacing the second amino acid L with W or Y exhibited similarly decreased editing efficiency inhibited by AdeI. Lastly, the substitution of the third amino acid V with A or L also demonstrated obvious inhibition. These findings underscored the importance of the first amino acid in PLV triplet for AdeI's proper recognition.

Additionally, we observed that the introduction of bulky amino acid residues such as W appeared to broaden the editing window near the PAM‐proximal position. For instance, A3A with the insertion of WLV, PYV, and PWV displayed ≈30% editing efficiency at position C_11_, whereas those with the insertion of PLV, PLL, and PLA exhibited editing efficiency below 20% at the same position (Figure S5g, Supporting Information).

The aforementioned results confirmed the hypothesis that AdeI could be employed to narrow the editing window of A3A_PLV_G25, and its inhibitory effect could be modified by adjusting the dosage or the amino acid composition of the insertion triplets.

### 2.4 Construction of more PICBEs with PLV Inserted at Diverse Positions

Considering such a minor change within the PLV triplet could lead to distinct editing outcomes (Figure S5d, Supporting Information), we further speculated that inserting PLV triplet into other positions in the loop1 of A3A might produce diverse editing outcomes. Therefore, we systematically inserted PLV triplet behind each amino acid in the loop1 of A3A and examined the editing behavior at the *HEK site2*, *HEK site3*, and *SiteA* target sites (Figure 2f). The results showed that A3A_PLV_K30 almost lost all activity, and A3A variants with PLV inserted behind I26, G27, R28, and H29 exhibited slight decreases in editing efficiency but achieved a significantly focused editing window. Among them, A3A_PLV_R28 demonstrated the best performance. For example, at the *HEK site2* locus, A3A exhibited editing efficiency of 23.2%, 18.5%, and 13.0% at C_4_, C_6,_ and C_11_, respectively, while A3A_PLV_R28 displayed that of 9.8%, 23.7%, and 0.2% at the same positions, showing a highly concentrated editing window.

It was evident that the insertion of a peptide downstream of each amino acid in loop 1 greatly influenced the editing outcome, likely due to significant changes in the microenvironment of A3A's substrate‐binding pocket. To further explore the modification potential of the PLV peptide on A3A, we inserted it into two other regions within the substrate‐binding pocket, loop 3 and loop 7, and tested them at *HEK site2*, *HEK site3*, and *SiteA* (Figure S6, Supporting Information). The results showed that the A3A variants with PLV peptide inserted in loop 3 exhibited slightly focused editing window and sufficient editing activity (Figure S6, Supporting Information), whereas those with insertions in loop 7 demonstrated higher activity loss alongside substantially enhanced editing precision. Notably, the PICBE variant A3A_PLV_D133 almost only edited one single base at *HEK site2* and *HEK site3*, showing extremely high editing precision. The subsequent comparison revealed that the A3A_PLV_D133's precision was 10.1‐fold of A3A (Figure 2g), although showing moderately decreased activity (Figure 2h).

From the comprehensive analysis of precision and activity (Figure 2g,h), we found that inserting peptides into different regions of A3A's substrate‐binding pocket could lead to markedly different effects on its editing behavior. Overall, inserting the PLV peptide into loop 7 brought the greatest precision enhancement but also resulted in an obvious loss of editing activity. Inserting the peptide into loop 1 also considerably improved precision while maintaining relatively high activity. However, inserting peptide into loop 3 had minimal effects on both precision and activity. Mapping these regions onto the structure of A3A (Figure 2i) revealed that loops 1 and 7 were located near the T_‐1_ position, upstream of the catalytic base C_0_ in the ssDNA substrate, while loop 3 was located near the G_1_ position, downstream of the catalytic base C_0_. This indicated that inserting the PLV peptide into regions closer to the N_0_ base was more likely to enhance editing precision which could be further improved by introducing single amino acid changes.

Overall, by inserting the PLV peptide into various loops within A3A's substrate‐binding pocket, we have developed a series of high‐precision A3A variants, such as A3A_PLV_R28 and A3A_PLV_D133, confirming the practicality of the peptide insertion strategy and provided a theoretical foundation for future modifications of other base editors.

### 2.5 Assessment of PICBEs’ off‐Target Effects

PICBEs showed obviously changed activity and precision, providing new tools for precise editing. However, while achieving target editing, base editors often induce off‐target effects which posed significant potential risks to cells. Generally, these off‐target editing could typically be classified into three types: 1) sgRNA‐dependent DNA off‐targeting: caused by the misrecognition of the spacer by the Cas protein‐sgRNA complex;^[^ ^2^ ^]^ 2) sgRNA‐independent DNA off‐targeting: caused by the random collision of free deaminase with ssDNA in the genomic DNA.^[^ ^8^ ^]^ 3) sgRNA‐independent RNA off‐targeting: caused by the erroneous binding of free deaminase to transcriptome.^[^ ^10^ ^]^

To investigate the sgRNA‐dependent DNA off‐targeting, we used Cas‐Offinder^[^ ^38^ ^]^ to predict the most likely off‐target site of *HEK site2*. Subsequently, through high‐throughput‐sequencing of the *HEK site2‐OT* amplicon, we observed that A3A exhibited 14.8% and 15.2% C‐to‐T conversion at C_4_ and C_6_, respectively, while A3A_PLV_G25 displayed 8.8% and 9.6% conversion at the same positions, suggesting that PLV insertion could decrease the sgRNA‐dependent DNA off‐targeting effecting (Figure S7, Supporting Information). Similar results were also observed in the comparison of A3A‐Y130F (0.60% at C_6_) and A3A‐Y130F_PLV_G25 (0.24% at C_6_). Noticeably, A3A‐Y132D_PLV_G25 exhibited only 0.06% conversion at C_6_, which was approximately equivalent to the background level (0.02%). Surprisingly, A3A_PLV_R28, which did not undergo any mutation introduction, displayed 0.07% conversion, indicating that the appropriate PLV insertion site could significantly decrease the off‐target effects of PICBEs.

Using whole‐genome sequencing to evaluate sgRNA‐independent DNA off‐targets is time‐consuming and expensive.^[^ ^39^ ^]^ Instead, a more economical and time‐saving detection method for assessing these effects has been reported. The orthogonal R‐loop assay has been proven to be effective for assessing sgRNA‐independent off‐target activity.^[^ ^40^ ^]^ In this assay, the orthogonal CRISPR system dSaCas9 was used to create ssDNA regions in human cells that acted as targets for the sgRNA‐independent deamination of base editors, which can be detected by amplicon sequencing.

**Figure 3:**
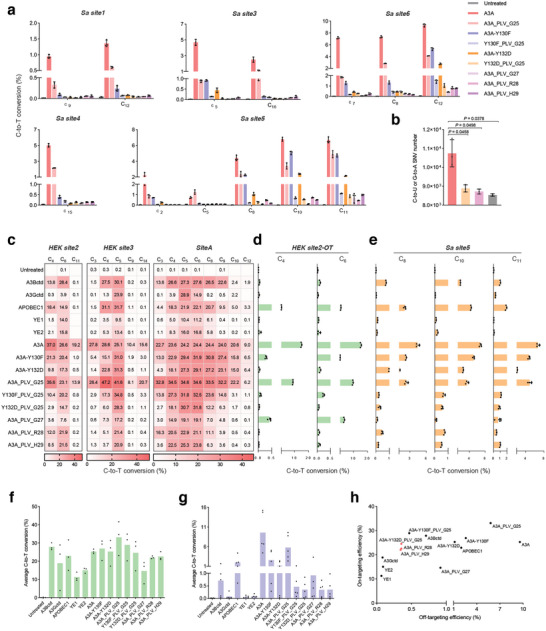
Comprehensive evaluation of PICBEs. a) The sgRNA‐independent off‐target editing frequency for PICBEs at five SaCas9 loci. b) RNA‐seq analysis of off‐target effects at the transcriptome for A3A, A3A‐Y132D_PLV_G25, and A3A_PLV_R28. c–e) On‐targeting (c), sgRNA‐dependent off‐targeting (d), and sgRNA‐independent off‐targeting (e) editing efficiency for hA3Bctd, hA3Gctd, mAPOBEC1, YE1, YE2, and PICBEs. f) Comparison of CBEs’ on‐target activity included *HEK site2*‐C_6_, *HEK site3*‐C_5_, and *SiteA*‐C_5_. g) Comparison of CBEs’ off‐target activity included *HEK site2‐OT*‐C_4_, C_6_ and *Sa site5*‐C_8_, C_10_, C_11_. h) Comprehensive comparison of CBEs's on‐taget and off‐target activity. All data came from three independent biological replicates. For heatmap, editing frequency higher than 0.1% are labeled in cells. Bars represent mean ± s.d. and *P* values are calculated using the two‐tailed Student's *t*‐test.

Subsequently, we conducted the R‐loop assay at five SaCas9 sites (**Figure** **3**a). A3A exhibited obvious sgRNA‐independent off‐target activity, which was only slightly alleviated by the mutation Y130F or Y132D. For example, A3A and variants with Y130F or Y132D mutations showed C‐to‐T conversion of 6.79%, 5.06%, and 2.39% at position C_10_ in *Sa site5*. In contrast, after inserting the PLV triplet behind G25, the above conversion decreased to 3.38%, 0.68%, and 0.55%. Besides, A3A with the PLV insertion behind G27, R28, and H29 only showed very low conversion of 0.10%, 0.72%, and 0.50%, respectively. This phenomenon indicated that PLV insertion could also decrease the sgRNA‐independent off‐targeting and could be further improved by introducing mutations, and similar results could be observed at all SaCas9 sites.

Through RNA‐seq, we comprehensively measured the number of SNVs (single nucleotide variants) at the transcriptome level for A3A‐Y132D_PLV_G25 and A3A_PLV_R28 showing high fidelity before. The analysis revealed that they both produced significantly decreased SNVs which approached the background level, compared to A3A, suggesting their high security (Figure 3b).

### 2.6 Comprehensive Comparison of PICBEs with other CBE Systems

To further evaluate the performance of PICBEs, we compared them with commonly used CBEs, including A3Bctd,^[^ ^37^ ^]^ A3Gctd,^[^ ^15^ ^]^ APOBEC1,^[^ ^3^ ^]^ YE1, and YE2.^[^ ^41^ ^]^ We selected *HEK site2*, *HEK site3*, and *SiteA* as the on‐targeting loci (Figure 3c). *HEK site2‐OT* and *Sa site5* were examined for sgRNA‐dependent or independent off‐target editing (Figure 3d,e).

At all target sites, APOBEC1 and A3Bctd showed a moderately narrowed editing window. In contrast, YE1, YE2, A3Gctd, and A3A‐Y132D_PLV_G25 showed significantly improved precision compared to A3A (Figure 3c). However, their activity varied across different sites. For example, A3Gctd showed only 3.9% C‐to‐T conversion at *HEK site2*‐C_6_ but achieved sufficient conversions of 23.9% and 28.9% at *HEK site3*‐C_5_ and *SiteA*‐C_5_, which was likely related to the motif preference of GC for A3Gctd. Besides, YE1 and YE2 showed obvious enhanced editing precision compared to APOBEC1, they both edited the above bases in low efficiency. Conversely, A3A‐Y132D_PLV_G25 showed that of 14.7%, 28.3%, and 30.7% at these positions. Through comprehensively analyzing the editing efficiency of *HEK site2*‐C_6_, *HEK site3*‐C_5_, *SiteA*‐C_5_, we noticed that the average activity of A3A‐Y132D_PLV_G25 was 0.97‐fold that of A3A, 1.30‐fold that of A3Gctd, 2.17‐fold that of YE1 and 1.63‐fold that of YE2, indicating that A3A‐Y132D_PLV_G25 could maintain stable activity in different target sites with diverse sequence compositions (Figure 3f).

Investigation of the *HEK site2‐OT* site revealed that YE1, YE2, A3Gctd, and A3A‐Y132D_PLV_G25 showed similarly low levels of sgRNA‐dependent off‐target editing, achieving 0.06%, 0.07%, 0.03%, and 0.06% C‐to‐T conversion at C_6_ (Figure 3d). Additionally, in R‐loop assay, they showed 0.04%, 0.09%, 0.19%, and 0.55% C‐to‐T conversion at *Sa site5*‐C_10_ (Figure 3e). Comparably, A3A exhibited that of 6.79% in the same position.

Further comprehensive comparison revealed that PICBEs, represented by A3A‐Y132D_PLV_G25, A3A_PLV_R28, and A3A_PLV_H29 could universally decreased the off‐target effects, meanwhile keeping sufficient on‐target activity (Figure 3f–h).

### 2.7 Construction of PIABEs

**Figure 4:**
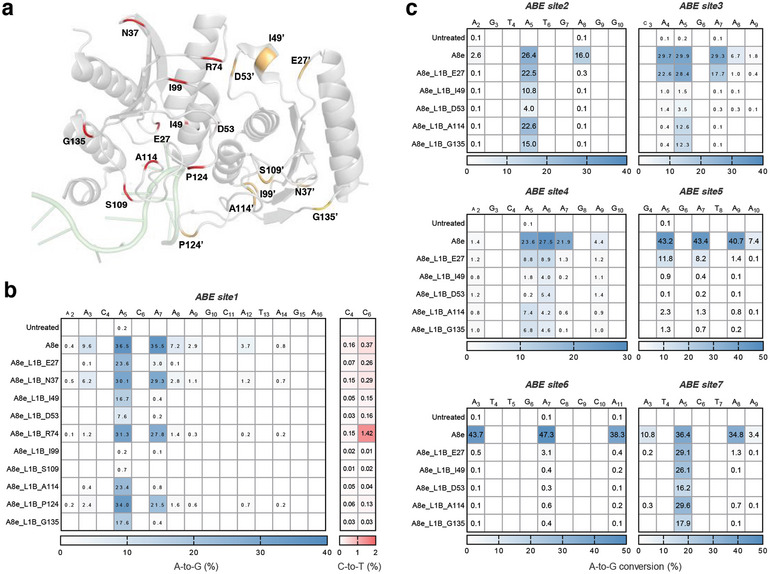
Construction of PIABEs. a) Structure of TadA‐8e (gray, PDB code 6vpc) in complex with ssDNA. The positions for L1B insertion are marked with red or yellow in each A8e monomer, and the ssDNA is marked in green. b) Editing efficiency of A8e with L1B inserted behind different amino acids. *ABE site1* was tested. c) A‐to‐G conversions of A8e with L1B inserted behind E27, I49, D53, A114, and G135. *ABE site2–site7* were tested. All data came from three independent biological replicates. For heatmap, editing frequency higher than 0.1% are labeled in cells.

**Figure 5:**
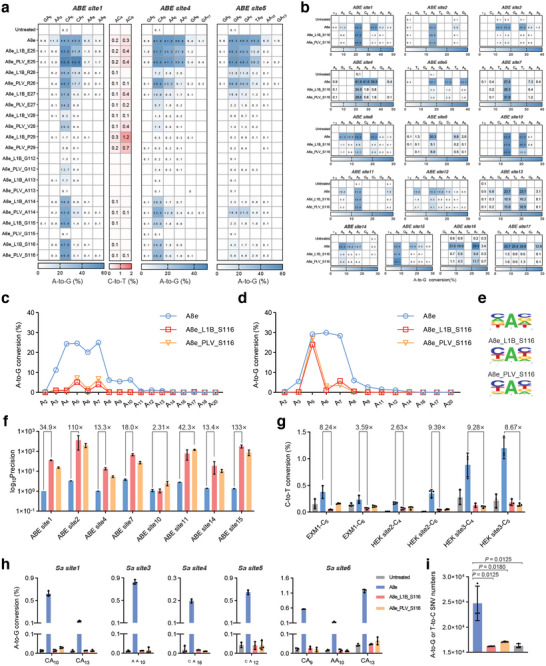
Further improvement of PIABEs. a) Editing efficiency of PIABEs at *ABE site1*, *ABE site4*, and *ABE site5*. b) A‐to‐G conversions of A8e and A8e_L1B/PLV_S116 at 16 endogenous sites containing all possible N_4_A_5_N_6_ motifs. c, d) Average editing efficiency of A8e and A8e_L1B/PLV_S116 at target sites with R_4_A_5_N_6_ (c) and Y_4_A_5_N_6_ (d) motifs. e) Logo sequence showing the motif preference of A8e and A8e_L1B/PLV_S116. A_0_ is referred to A_5_ in target sites. Frequencies shown in (b) are input. f) Precision comparison of A8e and A8e_L1B/PLV_S116 at target sites with Y_4_A_5_N_6_ motif. Precision is calculated using A_5_/A_N_ with the second high A‐to‐G conversions and the sites shown in (b) are included in statistics. g) C‐to‐T conversions of A8e and A8e_L1B/PLV_S116 at *EMX1*, *HEK site2*, and *HEK site3*. h) The sgRNA‐independent off‐target editing frequency for A8e and A8e_L1B/PLV_S116 at five SaCas9 loci. i) RNA‐seq analysis of off‐target effects at the transcriptome for A8e and A8e_L1B/PLV_S116. All data came from three independent biological replicates. For heatmap, editing frequency higher than 0.1% are labeled in cells. Bars represent mean ± s.d and *P* values are calculated using the two‐tailed Student's *t*‐test.

As mentioned above, we have successfully engineered PICBEs with high activity or accuracy. However, it remained unclear whether this strategy could be applicable in ABE system, another main type of BE. We decided to apply the strategy to engineer the TadA‐8e (A8e)‐derived ABE, the most active ABE system albeit with an extremely broad editing window.^[^ ^42^ ^]^ When analyzing the structure of A8e (Figure 5a), we observed that the active site was surrounded by a long, unstructured loop (from G105 to V130). Additionally, 50.3% (84/167) of A8e consists of loops, which is higher than the 41.9% (83/198) observed in A3A. Given this, we were concerned that the structural changes induced by the short PLV insertion might be overshadowed by the abundance of loops in A8e, we speculated that inserting a larger peptide would lead to more substantial changes. Given that the L1B insertion caused a significant reduction in the activity of PICBEs (Figure 1c,d), we ventured a risky hypothesis that L1B peptide insertion might have a dramatic impact on the function of A8e. Hence, we ultimately selected the L1B peptide and inserted it into substrate‐accommodated loops in A8e (**Figure** **4**a), as well as substrate‐distal loops for control and comparison, then testing their performance at *ABE site1* (Figure 4b).

The results showed that A8e achieved A‐to‐G conversion of 9.6%, 36.5%, and 35.5% at A_3_, A_5_, and A_7_, respectively. Conversely, PIABEs with L1B inserted behind I99 and S109 almost completely abolished the activity, possibly due to the disruption of correct folding. As expected, those with the L1B inserted at positions far from the active center, such as N37, R74, and P124, resulted in minimal impact on editing efficiency at A_5_. Notably, those variants with L1B inserted at positions near or inside the substrate‐binding pocket, such as E27, I49, D53, A114, and G135, led to significantly reduced A‐to‐G conversion at A_3_ and A_7_, while showing substantial conversion at A_5_, suggesting a significant narrow of the editing window and a huge improvement in precision.

In addition, a recent study reported that apart from A‐to‐G conversion, A8e could lead to C‐to‐T conversion at the same target site.^[^ ^21^ ^]^ We observed that most engineered PIABEs showed lower C‐to‐T conversion compared to A8e, which showed that of 0.4% at position C_6_. However, as an exception, the A8e_L1B_R74 exhibited 1.4% C‐to‐T conversion at the same position, which was 3.5‐fold that of A8e (Figure 4b).

To further examine the performance of the potentially precise PIABE variants, A8e_L1B_E27, I49, D53, A114, and G135, we selected an additional six genomic loci for testing. At *ABE site2* and *ABE site7*, A8e_L1B_E27 and A114 displayed almost no bystander editing, achieving focused editing at A_5_, yet A8e_L1B_I49, D53, and G135 exhibited insufficient editing efficiency despite maintaining similar precision (Figure 4c).

We also observed that A8e_L1B_E27/A114 had minimal activity at the *ABE site3* and *ABE site5* with context sequences of A_4_A_5_G_6_ and G_4_A_5_G_6_. As a contrast, the *ABE site1*, *ABE site2*, and *ABE site7*, at which PIABEs exhibited high editing activity, had motifs of C_4_A_5_T_6_, T_4_A_5_T_6_, and T_4_A_5_C_6_, respectively, indicating that PIABEs with L1B insertion seemed to preferentially edit A_5_ in the contexts rich in pyrimidines.

### 2.8 Improvement of PIABEs

Changing the compositions and embedded positions of inserted peptides profoundly influenced the performance of PICBEs (Figures 1d and 2k). Hence, to further improve the performance of A8e_L1B_E27/A114 and relieve their motif preferences, we finely inserted L1B or PLV fragments around E27 and A114. *ABE site1* (C_4_A_5_C_6_), *ABE site 4* (C_4_A_5_A_6_), and *ABE site 5* (G_4_A_5_G_6_) were selected for on‐target test (**Figure** **5**a).

Some outcomes caught our attention. A8e_PLV_E25 exhibited a 1.17‐fold higher average editing efficiency at A_5_ (51.6%) compared to A8e (44.1%) in the three target sites, and showed a narrower editing window with decreased off‐target activity in subsequent off‐target tests (Figures S8 and S9, Supporting Information). Considering A8e was one of the most active ABEs, such a 17% enhancement of activity caused by A8e_PLV_E25 indicated a substantial improvement. Coincidentally, the peptide insertion site of A3A_PLV_G25, which showed higher activity than A3A, was also the 25th residue (Figure 1c). Moreover, A8e_PLV_R26 showed similar editing efficiency at A_5_ compared to A8e, accompanied by decreased bystander editing, akin to the result of A3A_PLV_R28 (Figure 2f). Besides, like A3A_PLV_K30, A8e_PLV_P29 almost lost its activity. These findings demonstrated that inserting PLV triplet at loop1 of A8e yielded surprisingly similar results to A3A, suggesting that engineering the loop1 of deaminases may be a universal strategy to improve the activity and reduce the bystander effects of BEs (Figure 5a).

Engineering PIABEs originated from A8e_L1B_E27 showed some deficiency in precision, while those similar to A8e_L1B_A114 seemed to have higher precision. At *ABE site1*, A8e_L1B_G115, A8e_L1B_S116, and A8e_PLV_S116 exhibited editing efficiency of 45.6%, 47.9%, and 46.6% at A_5_, with extremely decreased bystander editing at A_3_ and A_7_.

Compared to A8e and A8e_L1B_A114, whose editing efficiency was 44.2% and 38.6% at A_5_, the three newly‐engineered PIABE variants almost retained full activity. Interestingly, the C‐to‐T conversions were obviously decreased caused by A8e_L1B/PLV_G112–S116, compared to A8e_L1B/PLV_E25–P29 (Figure 5a).

At *ABE site4*, A8e exhibited editing efficiency of 41.9%, 41.6%, and 38.8% at A_5_, A_6_, and A_7_, respectively. Conversely, A8e_L1B_A114 (20.0%, 1.7%, and 0.7%), A8e_L1B_S116 (24.9%, 1.9%, and 0.8%), and A8e_PLV_S116 (29.6%, 5.6%, and 1.8%) exhibited significantly improved accuracy, indicating that A8e_L1B/PLV_S116 could maintain more sufficient activity in pyrimidine‐purine contexts than A8e_L1B_A114. However, A8e_L1B_S116 and A8e_PLV_S116 exhibited editing efficiency of only 4.5% and 5.6% at A_5_ in ABE site5, suggesting that the editing activity of A8e_L1B/PLV_S116 in target sites with purine‐rich motif were still limited.

To unbiasedly investigate the motif preferences of A8e_L1B_S116 and A8e_PLV_S116, we tested them at a total of 16 target sites, including all possible context sequences around the position A_5_ (Figure 5b). The results were in accordance with the previous guess that A8e_L1B/PLV_S116 showed sufficient activity at pyrimidine‐rich motifs like *ABE site1* (C_4_A_5_C_6_) and exhibited low activity at purine‐rich motifs like *ABE site3* (A_4_A_5_G_6_). Further comprehensive statistics revealed that at those target sites containing R (A/G)_4_A_5_N_6_ motif, they only showed 20.7% and 29.2% activity compared to A8e (Figure 5c).

In contrast, at target sites containing Y (C/T)_4_A_5_N_6_ motif, they could maintain 82.2% and 92.8% activity compared to A8e at A_5_, and showed minimal bystander editing (Figure 5d). These results proved that the L1B and PLV insertion behind S116 endowed evident motif preference for Y_4_A_5_N_6_ compared to A8e which showed no obvious motif preference, which was further confirmed by the sequence logo plot (Figure 5e).

Additionally, we quantified the precision of the target sites with Y_4_A_5_N_6_ motif by calculating the A‐to‐G conversion of A_5_/A_N_ with the second high efficiency (Figure 5f). Detailed analysis revealed that A8e_L1B/PLV_S116 achieved up to 133‐fold improvement of precision compared to A8e. Notably, the precision of A8e_L1B_S116 at the eight sites shown in the Figure 5f was 2.34, 1.81, 2.51, 2.51, 0.42, 0.65, 1.78, and 2.1‐fold (with an average of 1.75‐fold) compared to A8e_PLV_S116. This suggested that the insertion of L1B, compared to PLV, moderately enhanced the precision of A8e.

Then, we tested A8e_L1B/PLV_S116 at several CBE sites to further investigate their ability to produce C‐to‐T conversion (Figure 5g). Compared to A8e, A8e_L1B/PLV_S116 showed greatly decreased C‐to‐T activity, from 2.63‐fold to 9.39‐fold. Subsequent off‐target tests for A8e_L1B/PLV_S116 revealed that their sgRNA‐dependent (Figure S10, Supporting Information) and sgRNA‐independent (Figure 5h) DNA off‐target editing activity were extremely low, which almost reached the background level. Similar results were observed in the RNA‐seq assay for detecting the sgRNA‐independent RNA off‐target effect (Figure 5i).

In conclusion, A8e_L1B/PLV_S116 can maintain high activity and maximized precision at target sites containing Y_4_A_5_N_6_ motif, with significantly decreased C‐to‐T side production and minimal off‐target effects in DNA and RNA‐level.

### 2.9 Comprehensive Comparison of PIABEs with other ABE Systems

Previous research reported the V82G,^[^ ^43^ ^]^ V106W,^[^ ^23^ ^]^ F148A,^[^ ^24^ ^]^ or N108Q/L145T (ABE9)^[^ ^25^ ^]^ mutation could improve precision and reduce off‐target effects of A8e. Subsequently, we compared them to the engineered PIABEs with high accuracy constructed in this work. *ABE site1* (C_4_A_5_C_6_), *ABE site4* (C_4_A_5_A_6_), and *CBE site10* (G_4_A_5_G_6_) were selected for on‐target test. *ABE site1‐OT* was examined for sgRNA‐dependent off‐target editing, and *Sa site5* was investigated for R‐loop assay‐based sgRNA‐independent off‐target editing.

**Figure 6:**
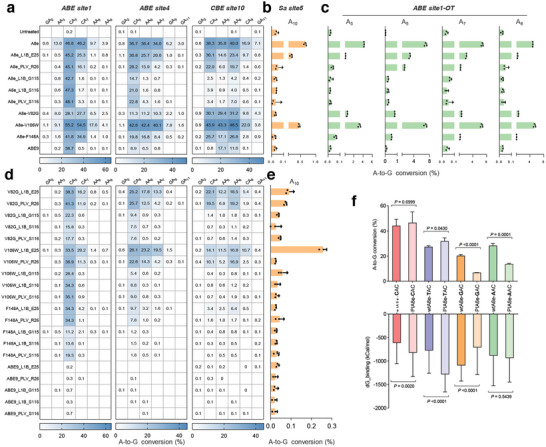
Comprehensive evaluation of PIABEs. a–c) On‐targeting (a), sgRNA‐dependent off‐targeting (b), and sgRNA‐independent off‐targeting (c) efficiency for A8e with V82G, V106W, F148A, N108Q/L145T (ABE9) and PIABEs. d, e) On‐targeting (d) and sgRNA‐independent off‐targeting (e) efficiency for A8e variants with mutations and L1B/PLV insertions. f) A‐to‐G conversions and dG_binding energy of A8e or A8e_PLV_S116 (PIA8e) with ssDNA containing **N_4_** A_5_C_6_ or **N_‐1_** A_0_C_1_ motif (A_5_ and A_0_ were referred to edited A). Molecular dynamics simulations of 100 ns are performed and the binding energy is calculated from the simulation trajectory with a step size of 1 ns. All data came from at least three independent biological replicates. For heatmap, editing frequency higher than 0.1% are labeled in cells. Bars represent mean ± s.d and *P* values are calculated using the two‐tailed Student's *t*‐test.

At the *ABE site1* locus, A8e‐V82G showed a significant decrease in editing efficiency, with no obvious change in the editing window (**Figure** **6**a). A8e‐V106W exhibited sufficient activity similar to A8e. A8e‐F148A displayed an editing efficiency of 41.8% at A_5_, equivalent to nearly 90% of A8e's efficiency (46.8%). Additionally, A8e‐F148A showed editing efficiency of 1.6% and 1.4% at A_3_ and A_8_, which was much lower than A8e's (13.0% and 9.7%). However, its bystander editing at A_7_ remained at 34.9%. Overall, the behavior of A8e‐F148A was similar to A8e_L1B_E25 or A8e_PLV_R26, which maintained considerable activity at primary positions like A_5_ and A_6_, while achieving a modest narrow in the editing window.

ABE9, as the latest generation of highly precise ABE system, exhibited an almost minimal editing window of A_5_–A_6_.^[^ ^25^ ^]^ It showed an editing efficiency of 38.7% at A_5_, equivalent to 82.7% of A8e's efficiency. Meanwhile, its bystander editing at A_7_ was only 0.5%. Compared to ABE9, A8e_L1B_S116 exhibited a higher editing efficiency of 47.3% at A_5_, while showing a similar editing efficiency of 1.7% at A_7_.

At the *ABE site4*, the performance of A8e with V82G, V106W, or F148A was similar to that at the *ABE site1* (Figure 6a). A8e exhibited A‐to‐G conversion of 31.8%, 31.9%, and 29.8% at A_5_, A_6_, and A_7_, respectively. ABE9 showed editing efficiency of 8.9%, 6.5%, and 0.8% at these positions, respectively. In contrast, A8e_L1B_S116 showed editing efficiency of 21.0%, 1.6%, and 0.8%, respectively, indicating that it achieved higher activity and precision than ABE9 at this locus.

At the *CBE site10* locus, A8e displayed editing efficiency of 38.3%, 35.8%, and 40.0% at A_4_, A_5_, and A_6_, respectively. Conversely, ABE9 showed that of less than 1% at A_4_, while its editing efficiency at A_5_ and A_6_ were 17.1% and 11.6%, respectively, equivalent to 47.8% and 29.0% of A8e's efficiency, demonstrating a stable activity and an effectively narrowed editing window (Figure 6a). However, similar to the editing outcome at the *ABE site5* (Figure 5a), A8e_L1B_S116 showed an editing efficiency of less than 2% at A_5_, indicating an obvious resistance to purine‐rich context.

In summary, the mutation V82G or V106W did not narrow the editing window of A8e, likely due to their primary design aimed at reducing RNA off‐target effects. Moreover, the variant A8e‐F148A, similar to A8e_L1B_E25 or A8e_PLV_R26, demonstrated a modestly narrowed editing window. ABE9 demonstrated stable activity and high precision across all targeted sites, although it exhibited a decrease in activity at pyrimidine‐rich contexts. Interestingly, both A8e_L1B_S116 and A8e_PLV_S116 showed great activity and precision in pyrimidine‐rich motifs. For example, we calculated the proportion of cells containing only A_5_‐to‐G_5_ conversion after editing with A8e and its mutants (Figure S11, Supporting Information). The average proportions of edited cells with only A_5_‐to‐G_5_ conversion were 0.49%, 37.28%, and 43.98% for A8e, ABE9 (A8e‐N108Q+L145T), and A8e_L1B_S116, respectively. This means that the A8e_L1B_S116 mutant constructed in this study showed 89.8‐fold and 1.2‐fold higher chances of obtaining the cell colony harboring only the A_5_‐to‐G_5_ conversion, compared to A8e and ABE9, respectively. Meanwhile, at the *ABE site4* locus, the numbers were 68.2‐fold and 5.2‐fold higher, meaning that A8e_L1B_S116 was more precise than ABE9 at the appropriate targets.

Furthermore, we examined the off‐target activity of these ABEs. At *Sa site5* locus, the A‐to‐G conversions were 0.65%, 0.45%, and 0.18% for A8e, V106W, and A8e_L1B_E25, respectively, while the efficiency of the other variants were all less than 0.1% (Figure 6b). At *ABE site1‐OT* locus, A8e, V106W, A8e_L1B_E25, and A8e_PLV_R26 exhibited editing efficiency of 7.1%, 7.0%, 3.0%, and 1.6% at A_5_, while the editing efficiency of the other highly precise A8e variants were all less than 1% (Figure 6c). These results indicated that ABE9 and A8e_L1B/PLV_S116 exhibited extremely low off‐target activity, showing significant high security

### 2.10 Improvement of Peptide Inserted ABE8e Through Amino Acid Changes

Given that the PLV insertion was compatible with Y132D mutation during engineering the A3A‐CBE, we attempted to insert L1B/PLV fragments into A8e with mutations V82G, V106W, F148A, or N108Q/L145T (Figure 6d). Notably, all variants with L1B/PLV insertion showed a great loss of activity at the *CBE site10* (G_4_A_5_G_6_), meaning that the motif preference for Y_4_A_5_N_6_ dominated the entire base editing behavior.

At the *ABE site1* locus (C_4_A_5_C_6_ motif), peptide insertion and single amino acid change showed a significant cumulative effect on the activity and precision of A8e. For example, A8e‐F148A showed editing efficiency of 41.8% and 34.9% at A_5_ and A_7_, and A8e_PLV_R26 showed that of 45.1% and 16.1% (Figure 6a). With the PLV insertion behind E25, A8e‐F148A_PLV_R26 showed single‐resolution‐level A‐to‐G conversion at A_5_ of 34.3%, with significantly decreased bystander editing of 1.1% at A_7_ (Figure 6d). Conversely, A8e showed 46.8% and 46.2% at the same positions. Hence, an obvious rank of activity and precision appeared. First, A8e owned the highest activity and the lowest precision. Second, A8e‐F148A and A8e_PLV_R26 showed slightly reduced activity and improved precision. Third, A8e‐F148A_PLV_R26 showed the lowest activity but the best precision. Similar regularity could be observed in *ABE site4* (C_4_A_5_A_6_) and PIABEs with V82G and V106W mutation.

It is worth noting that ABE9 lost almost all activity at the three target sites after inserting the L1B/PLV fragments. Considering ABE9 performed not well in pyrimidine‐rich contexts and A8e_L1B/PLV_S116 performed not well in purine‐rich contexts, we hypothesized the additive effect of selectivity finally resulted in the loss of activity of ABE9‐derived PIABEs.

Moreover, almost all tested PIABE variants with amino acid changes exhibited sgRNA‐independent off‐target efficiencies below 0.1% (Figure 6e), demonstrating that the reduction in off‐target effects was cumulative through the combined effects of amino acid changes and peptide insertions.

### 2.11 Molecular Dynamics Simulation Revealed the Possible Mechanism for Changed Motif Preference

As mentioned above, A8e showed no motif preference, while A8e_PLV_S116 showed almost completely open/closed activity in Y_4_A_5_N_6_ and R_4_A_5_N_6_ motifs, respectively (Figure 5c,d). So far, no study has reported such a significant change in motif preference of base editors. To uncover the possible mechanism behind this phenomenon, we conducted a series of molecular dynamics simulations.

As original input, A8e monomer with ssDNA (TGTTCC_‐1_A_0_C_1_TT) was retained from the ABE8e complex (PDB code 6vpc). Through *in silico* mutation, A8e or A8e_PLV_S116 with ssDNA containing four motifs, C_‐1_A_0_C_1_, T_‐1_A_0_C_1_, A_‐1_A_0_C_1_, and G_‐1_A_0_C_1_ were obtained (A_0_ referred to the edited base). After a 100 ns molecular dynamics simulation using GROMACS software, the dG_binding energy between deaminase and ssDNA was calculated. Compared to A8e, A8e_PLV_S116 showed significantly enhanced binding energy with ssDNA containing C_‐1_A_0_C_1_ or T_‐1_A_0_C_1_ motif, and significantly reduced binding energy with those containing G_‐1_A_0_C_1_ motif (Figure 6f). Considering that *ABE site1*, *site7*, *site8*, and *site9* contained corresponding motif of C_4_A_5_C_6_, T_4_A_5_C_6_, A_4_A_5_C_6_, and G_4_A_5_C_6_ (A_5_ referred to the edited base) (Figure 5b), we attempted to associate the binding energy change with the editing efficiency change. Remarkably, if the binding energy was enhanced (for C_‐1_A_0_C_1_ and T_‐1_A_0_C_1_ motif), the editing efficiency also increased, while, if the binding energy was reduced or unchanged (for A_‐1_A_0_C_1_ and G_‐1_A_0_C_1_ motif), the editing efficiency concomitantly decreased, proving the change of motif preference was indeed caused by the change of binding energy.

However, as the smallest insertion unit tested in this work, the PLV triplet, which were all non‐polar amino acids, had a negligible contribution to the binding energy in the complex of A8e_PLV_S116 with ssDNA containing N_‐1_A_0_C_1_ motifs (Figure S12, Supporting Information), in accordance with the predicted structure of A8e_PLV_S116, in which the PLV triplet was far away from the ssDNA (Figure S13, Supporting Information).

Hence, we hypothesized that due to the enhanced flexibility and oscillation of the PLV triplet‐containing loop, the extended loop would be more likely to approach the ssDNA. The inserted PLV triplet could then generate steric hindrance against larger purine bases, thereby facilitating the entry of smaller pyrimidine bases into the binding pocket and catalytic center of A8e_PLV_S116. This spatial selection mechanism ultimately accounts for the strong preference toward the Y_4_A_5_N_6_ motif.

## 3 Discussion

Genetic diseases are characterized by significant harm, difficulty in treatment, and often a shortage of therapeutic methods, resulting in prolonged treatment cycles, which extremely increase the burden on patients.^[^ ^44^ ^]^ According to statistics, most of observed human genetic variants are SNPs, with over 99% currently lacking a clinical interpretation,^[^ ^45^ ^]^ theoretically most of which can be corrected by base editors.^[^ ^4^ ^]^ Initial versions of base editors often suffered from insufficient editing efficiency, low editing accuracy, and high off‐target effects.^[^ ^3^, ^4^ ^]^ Therefore, researchers frequently engineered them to improve their performance. Through directed evolution^[^ ^42^ ^]^ or rational design,^[^ ^22^ ^]^ highly performing base editors have been developed, often with one or several mutations near the active site of the deaminase. However, conventional amino acid changes have become increasingly saturated and can not fully unleash the catalytic potential of deaminases, necessitating more effective and universal methods to improve base editors.

Here, we systematically inserted peptide fragments into cytidine deaminase and adenine deaminase, resulting in A3A_PLV_G25 and A8e_PLV_E25 with higher editing efficiency than A3A and A8e, as well as A3A_PLV_R28, A3A_PLV_D133, A3A‐Y132D_PLV_G25, and A8e_L1B/PLV_S116, which showed high editing accuracy and minimal off‐target effects in DNA and RNA‐level. We believe that the high‐activity and high‐precision PIBEs developed in this study can exert powerful applications in both eukaryotes and prokaryotes.

In eukaryotes, base editors can efficiently generate SNP‐associated disease models^[^ ^46^, ^47^ ^]^ and correct genetic diseases‐related SNPs^[^ ^48^ ^]^. According to ClinVar database, 14% and 47% of known pathogenic human SNPs can theoretically be treated by CBE or ABE,^[^ ^3^ ^]^ respectively. However, most SNPs are located in genomic regions surrounded by other editable bases, leading to unwanted bystander editing with base editors. Given that the genetic diseases‐related SNPs are often located in a protein‐coding gene, the bystander editing may result in non‐silent mutations that alter the amino acid sequence, acting as severe side effects. What is more concerning is that recent studies have reported that silent mutations in eukaryote genes are often strongly non‐neutral,^[^ ^49^, ^50^ ^]^ possibly because silent mutations in a gene can affect the degradation rate of the encoded mRNA, thereby influencing the concentration of mRNA within the cell. Therefore, whether bystander editing leads to silent or non‐silent mutations, both can potentially have a negative impact on the encoded protein, which undoubtedly raises concerns about the safety of using base editors for disease treatment. Hence, this places new high demands on the accuracy of base editors. In this context, the precise PIBEs developed in this study can maintain high editing efficiency and a focused editing window simultaneously, thus potentially significantly reducing harmful bystander editing while preserving their promising therapeutic effects in treating SNP‐mediated genetic diseases.

In prokaryotes, base editors can achieve: 1) rapid gene silencing by converting the “CGA” codon to “TGA” stop codon;^[^ ^51^ ^]^ 2) targeted gene mutagenesis by expressing multiple sgRNAs directed to a specific gene;^[^ ^52^ ^]^ 3) mutagenesis in large DNA fragment by fusing deaminases to RNA polymerase^[^ ^53^ ^]^ or helicases.^[^ ^54^ ^]^ In fact, all of these applications rely on highly efficient deaminases. Hence, the high‐activity deaminases and derived PIBEs developed in this study could achieve high performance in bacterial applications.

Clearly, the peptide insertion strategy effectively enhanced the performance of base editors and showed good universality. Through extensive investigation, this strategy has shown some regularities. First, the optimal position for peptide insertion was located around the active site pocket of the deaminase and near the N_‐1_ base in ssDNA substrate. Secondly, small‐sized fragments, such as PLV, are recommended for the initial selection and testing, while careful extension of the insertion length may lead to a more substantial impact. Once the loop for insertion was selected, it was recommended to insert peptides behind every amino acid in this loop, as even a difference of just one amino acid position could result in vastly different changes in activity and accuracy.

From the results, it was evident that the insertion of peptides significantly altered the motif preference of A3A and A8e (Figures 1i and 5e). For example, the insertion of L1B/PLV in this study primarily enhanced the selectivity A8e toward motifs rich in pyrimidines. Through molecular dynamics simulation, this phenomenon was confirmed to be achieved through two mechanisms: by enhancing binding to ssDNA containing Y_4_A_5_N_6_ motifs and reducing the affinity for those with R_4_A_5_N_6_ motifs. During the development of ABE system, TadA‐6.3 and TadA‐7.10 exhibited slight preference of YAN.^[^ ^3^ ^]^ However, their editing windows were broader and their motif preferences were weaker, compared to A8e_PLV/L1B_S116. Additionally, considering that the side chain of PLV did not carry obvious charges, it was possible that PLV produced steric hindrance against larger purine bases through a volume gating effect, thereby excluding the R_4_A_5_N_6_ motif. Therefore, if other amino acid compositions, such as highly charged peptide fragments, were inserted, they might exhibit greater affinity for purine‐rich motifs. Considering that the insertion of PLV/L1B improved the editing precision of A8e to approach the single‐base level, it was imperative to construct sufficiently large peptide insertion libraries to screen those peptide fragments that could provide diverse selectivity, thus enriching the editing breadth of precise base editors.

We also found that peptide insertion at position R74 resulted in more C‐to‐T conversions for A8e. This result was similar to the finding that mutation of Y73 located in A8e dimer interface could significantly enhance the C‐to‐T activity of TadDE,^[^ ^55^ ^]^ indicating that engineering around Y73 and R74 could help develop and improve A8e‐based CBEs (Figure 4a). Additionally, a recent report introduced several amino acid changes in D133 residue of A3A, slightly narrowing the editing window.^[^ ^21^ ^]^ By contrast, the PICBE variant A3A_PLV_D133 showed significantly focused editing window of near 1–2 bases. In addition, by comparing the PIABE with TadA‐based ABE system based on amino acid mutations,^[^ ^3^, ^22^, ^24^, ^56^, ^57^ ^]^ we found that almost all previous studies overlooked the G115 to S116 residues (Figure S14, Supporting Information). However, we confirmed that inserting peptides behind these residues were critical for narrowing the editing window and enhancing the precision (Figure 5a). These findings suggest that the one‐step peptide insertion strategy is more efficient than the traditional amino acid change strategy in identifying residues that significantly affect editing behavior, as the latter requires considering 19 possible amino acid types when mutating a single residue.

Furthermore, we recognized that the insertion strategy could synergistically work with the amino acid change strategy, and their effects were generally cumulative, implying that in the practical development of high‐precision base editors, it was essential to explore more combinations of them to achieve optimal variants. Additionally, during the preparation of this manuscript, Yang and colleagues published several mutations that outperform the existing Y130F and Y132D mutations for A3A, such as Y130A or Y130G.^[^ ^21^ ^]^ Therefore, we have reasoned that combining A3A_PLV_G25 with these newly developed single amino acid changes could yield a higher precise A3A‐CBE while maintaining sufficient on‐target activity.

We also noticed that the high‐precision PICBEs, such as A3A_PLV_R28, can still modestly introduce unwanted mutations at both target and off‐target loci (Figure 3d,e). We hypothesize that the inserted PLV peptide may not be robust enough to remodel the highly flexible loop regions that constitute the ssDNA binding pocket. Given that the A8e mutants with insertions of the longer L1B peptide showed moderately improved precision compared to those with the shorter PLV peptide, we speculate that a more precise PICBE could be developed by stepwise prolonging the inserted peptide. However, considering that A3A_L1B_G25 has lost its activity (Figure 1d), a more rigorous investigation of inserted peptide length and composition is needed. Additionally, to further improve the accuracy and reduce the off‐target effects in the future, we could split the peptide‐inserted deaminases and incorporate them into a chemical^[^ ^58^, ^59^ ^]^ or light‐induced^[^ ^6^ ^]^ dimerization system. These could then be fused with a high‐fidelity Cas protein^[^ ^60^, ^61^ ^]^ and an engineered sgRNA^[^ ^62^ ^]^ to enhance editing precision.

Besides, other cytidine deaminases such as APOBEC1, A3B, A3G, and the DddA deaminase^[^ ^63^ ^]^ which acts on double‐stranded DNA, are also part of excellent CBE systems, sharing similar structural compositions with A3A. Considering that even A8e, with relatively low homology with A3A, can adapt to the insertion strategy, it is highly likely that these deaminases could also accommodate insertions of peptides. Recently, an increasing variety of base editors have been developed, such as the CGBE (C‐to‐G)^[^ ^64^, ^65^ ^]^, AYBE (A‐to‐Y)^[^ ^66^ ^]^, gGBE (G‐to‐Y)^[^ ^67^ ^]^, and TBE (T‐to‐G/C).^[^ ^68^, ^69^ ^]^ Considering that they both possess DNA binding pockets, the insertion of peptide fragments like PLV/L1B could potentially alter their substrate recognition preferences and enhance their editing precision. Moreover, strict motif preferences for Y_4_A_5_N_6_ of A8e_PLV/L1B_S116 act as a double‐edged. On the one hand, it promotes maximized precise editing for those target sites with appropriate motifs. On the other hand, in many cases, the desired adenine is not located at the N_5_ position during a protospacer even though it is evolved in a YAN motif. Fortunately, an increasing number of Cas proteins that circumvent PAM restrictions have been developed, such as SpCas9‐NG (NGN PAM),^[^ ^70^ ^]^ SpRY (near‐PAMless),^[^ ^71^ ^]^ and eNme2Cas9 (N_4_CN PAM).^[^ ^72^ ^]^ Coupled with them, A8e_PLV/L1B_S116 could exhibit strong potential in precise therapy.

## 4 Experimental Section

### Plasmid Cloning

The DNA fragments of rAPOBEC1, hA3A, hA3Bctd, hA3Gctd, TadA‐8e, and AdeI were synthesized by GenScript. After amplification using Phanta Max Super‐Fidelity DNA polymerase (Vazyme), these fragments were ligated to the backbone of pCMV‐BE3 (Addgene #73021) under the CMV promoter using the Hieff Clone One Step Cloning Kit (YEASEN). The introduction of point mutations of peptide fragments was performed using Mut Express II Fast Mutagenesis Kit V2 (Vazyme). Protospacer oligos shown in Tables S1 and S2 (Supporting Information) were phosphorylated by PNK (Thermo Fisher Scientific) and cloned into BsaI‐digested sgRNA expression plasmids by T4 DNA Ligase (Thermo Fisher Scientific). All insertion positions of PIBEs constructed in this study were shown in Table S3 (Supporting Information).

### Culture Condition and Transfection

HEK293T (ATCC CRL‐3216) cells were kept in Dulbecco's modified Eagle medium (DMEM, Gibco) supplemented with 10% (vol/vol) fetal bovine serum (FBS, Gibco) in 5% CO_2_ incubator (Crystal) at 37°C.

HEK293T cells were seeded into 48‐well plates (Corning) at a density of 55 000 cells per well and were transfected 18–24 h after plating. Transfection was performed using Lipofectamine 3000 (Invitrogen) according to the manufacturer's instructions. For on‐target genomic editing, 500 ng base editor plasmids and 200 ng sgRNA expression plasmids were co‐transfected. For orthogonal R‐loop assays, 300 ng SpCas9‐containing plasmids, 200 ng SpCas9 sgRNA expression plasmids, 300 ng dSaCas9‐containing plasmids, 200 ng SaCas9 sgRNA expression plasmids were co‐transfected.

### Targeted Amplicon Sequencing

For next‐generation sequencing, PCR amplification was performed using primers synthesized with different barcodes. PCR products were verified on 2% agarose gel and purified using the Universal DNA Purification Kit (Tiangen Biotech). Amplicons with various barcodes were mixed and sequenced on an Illumina MiSeq instrument according to the manufacturer's protocols (GENEWIZ). The quality of raw sequencing reads was evaluated using Fastp,^[^ ^73^ ^]^ and those with a quality score below 15 were discarded. Adapters were trimmed using PANDAseq,^[^ ^74^ ^]^ and Fastq‐multx^[^ ^75^ ^]^ was used to demultiplex the paired sequences. CRISPResso2^[^ ^76^ ^]^ was used to calculate the editing efficiency.

For Sanger sequencing, the target regions were PCR amplified and then analyzed by Beijing Tsingke Biotech Co., Ltd. The results were quantified using EditR.^[^ ^77^ ^]^

### Off‐Target Amplicon Sequencing

The sgRNA‐dependent off‐target sites were investigated using Cas‐OFFinder.^[^ ^36^ ^]^ The sgRNA‐independent off‐target sites were consistent with dSaCas9 targeting sites in the orthogonal R‐loop assays. Next‐generation sequencing was performed as described above. CRISPResso2 was used to calculate the editing efficiency of off‐target sites.

### Analysis of RNA Off‐Target Editing

Total RNA was extracted using the Trizol (Invitrogen), and the RNA purity was monitored by NanoDrop 2000 spectrophotometer (NanoDrop Technologies) and a Bioanalyzer 2100 system (Agilent Technologies). RNA contamination was assessed by 1.5% agarose gel.

The mRNA was purified from the total RNA using poly‐T oligo‐attached magnetic beads. Sequencing libraries were generated from the purified mRNA using the VAHTS Universal V6 RNA‐seq Library Kit for MGI (Vazyme) following the manufacturer's recommendations with unique index codes. The library quantification and size were assessed using Qubit 3.0 Fluorometer (Life Technologies) and Bioanalyzer 2100 system (Agilent Technologies). Subsequently, sequencing was performed on a MGI‐SEQ 2000 platform by Frasergen Bioinformatics Co., Ltd. (Wuhan, China).

SOAPnuke (v2.1.0) was used to filter the sequencing data. HISAT2 (v2.1.0) was used to compare clean reads with the reference genome, and Bowtie2 (v2.3.5) was used to align the quality‐controlled sequence to the reference transcription sequence. After using a Picard (v1.96) tool suite to sort the bam file obtained by comparing it with the reference genome and remove duplication, SAMtools (v0.1.19) combined with GATK (v3.7) method were conducted to call for obtaining high‐quality SNV information. The numbers of all 12 types of mutations were included in statistics and tabulated.

### Structure Construction

The complex structure of TadA‐8e with ssDNA (TGTTCC_‐1_A_0_C_1_TT) was obtained from the cryo‐EM structure of ABE8e (PDB code 6vpc). SWISS‐MODEL^[^ ^78^ ^]^ was used to predict the structure of A8e_PLV_S116. The base_‐1_ in the binding ssDNA was mutated using PyMOL (The PyMOL Molecular Graphics System, Schrödinger, LLC).

### Molecular Dynamics Simulation

All Molecular dynamics simulations were carried out using GROMACS. The topology and coordinate files were generated using the *pdb2gmx* program with parameters from the amber_14_sb_parmbsc1^[^ ^79^, ^80^ ^]^ force field. The complex was placed in the center of a cubic box with an edge length of ≈1 nm from the protein surface to the box boundary and then solvated with SPC216 water molecules. Specific numbers of Na^+^ and Cl^−^ ions were added to the system to neutralize the complex charge. To optimize the system, energy minimization was performed until the maximum force <1000 kJ mol^−1^ nm^−1^. The system was then heated to 310 K for 100 ps with a time step of 2 fs by constant NVT equilibration. Subsequently, constant NPT simulations of 500 ps were performed to equilibrate the system at a pressure of 1.0 bar. Finally, production molecular dynamics simulations of 100 ns were performed for the following analyses. The binding energy between A8e variants and ssDNA along the simulation trajectory was calculated using the *gmx_mmpbsa* script with a step_size of 1 ns.

### Statistics Analysis

Statistical analyses were conducted using GraphPad Prism 9 (GraphPad Software). Data are presented as mean ± s.d. Unpaired two‐tailed Student's *t*‐tests were used to determine the *P* values. *P* < 0.05 was considered significant. Online software WebLogo was used to form the sequence logo plot.

## Conflict of Interest

The authors declare no conflict of interest.

## Author Contributions

Q.C. designed and carried out experiments, analyzed data, and wrote the manuscript. Y.S., J.Y., Y.L., R.Q., F.Z., and Z.D. discussed and revised the manuscript. Y.S. conceived the study, analyzed the data, and wrote the manuscript.

## Supporting information



Supporting Information

Supporting Information

## Data Availability

The data that support the findings of this study are available in the supplementary material of this article.
