# Supplementary material for: Engineering of Peptide‐Inserted Base Editors with Enhanced Accuracy and Security
Source: Small. 2025 Feb 25;21(14):2411583. doi: 10.1002/smll.202411583 (PMC11983243; doi:10.1002/smll.202411583)
Supplement: Supplementary file 1 — Supporting Information [file SMLL-21-2411583-s002.pdf]

NANO · MICRO
small

Supporting Information

for *Small*, DOI 10.1002/smll.202411583

Engineering of Peptide-Inserted Base Editors with Enhanced Accuracy and Security

*Qi Chen, Yangning Sun, Jia Yao, Yingfan Lu, Ruikang Qiu, Fuling Zhou, Zixin Deng and Yuhui Sun**

NANO MICRO
small

Engineering of peptide-inserted base editors with enhanced accuracy and security

Qi Chen, *Yangning Sun*, *Jia Yao*, *Yingfan Lu*, *Ruikang Qiu*, *Fuling Zhou*, *Zixin Deng*, *and Yuhui Sun**

Q. Chen, Y. Sun, J. Yao, Y. Lu, R. Qiu, F. Zhou, Z. Deng, Y. Sun
Department of Hematology
Zhongnan Hospital of Wuhan University
School of Pharmaceutical Sciences
Wuhan University
Wuhan, 430071, China
E-mail: yhsun@whu.edu.cn

Y. Sun
School of Pharmacy
Huazhong University of Science and Technology
Wuhan, 430030, China

Q. Chen, Y. Sun, J. Yao, Y. Lu, R. Qiu, F. Zhou, Z. Deng, Y. Sun
Key Laboratory of Combinatorial Biosynthesis and Drug Discovery (Ministry of Education)
Wuhan University
Wuhan, 430071, China

Table of Contents

Supporting Figures

Figure S1. On-target editing efficiency for A3A and A3A_PLV_G25.

Figure S2. C-to-T conversions for point mutated A3A and A3A_PLV_G25.

Figure S3. Comparison of base editing precision of A3A and PICBE in RNF2 loucs.

Figure S4. C-to-T conversions for A3A and A3A_PLV_G25 with AdeI at different doses.

Figure S5. C-to-T conversions for A3A_PLV_G25 with AdeI at different doses.

Figure S6. Test of PICBEs with PLV inserted in various regions.

Figure S7. Comparison of sgRNA-dependent off-target effects of PICBEs.

Figure S8. Comparison of the on-target activity of A8e and A8e_PLV_E25.

Figure S9. Comparison of the sgRNA-independent off-target effects of A8e and A8e_L1B/PLV_E25.

Figure S10. Comparison of sgRNA-dependent off-target effects of PIABEs.

Figure S11. Comparison of base editing precision of A8e, ABE9, and PIABE.

Figure S12. Binding energy for PLV triplet.

Figure S13. Structure comparison of A8e and A8e_PLV_S116.

Figure S14. Comparison of TadA-based ABEs developed by different engineering methods.

Supporting Tables

Table S1. Target sgRNA-protospacer sequences in this study.

Table S2. Off-target sgRNA-protospacer sequences in this study.

Table S3. Inserted peptide sequences for PIBEs.

Supporting Sequences

Plasmids (functional amino acid sequences shown only) for HEK293T in this study.

Supporting Figures

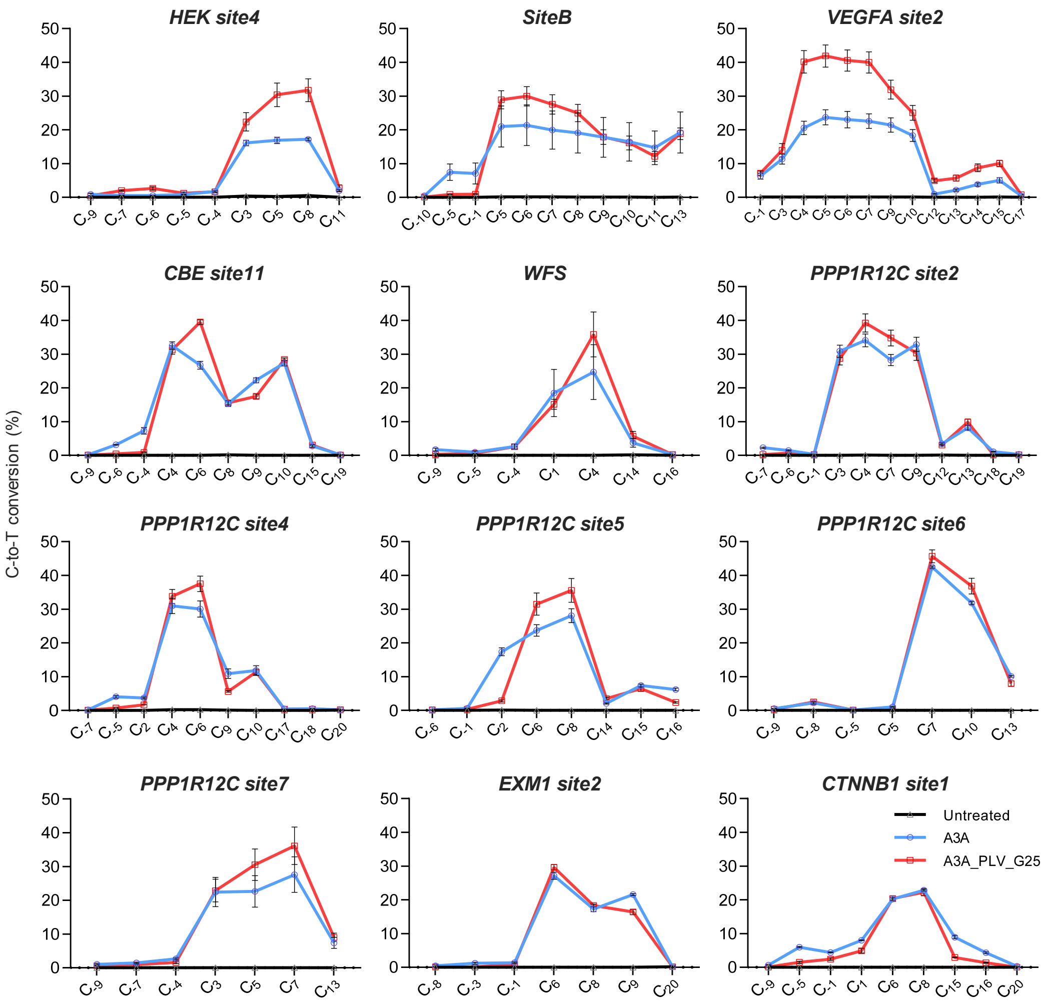

Figure S1. On-target editing efficiency for A3A and A3A_PLV_G25. Twelve endogenous target sites were tested. All data are shown as individual data points and means ± s.d. for n = 3 independent biological replicates.

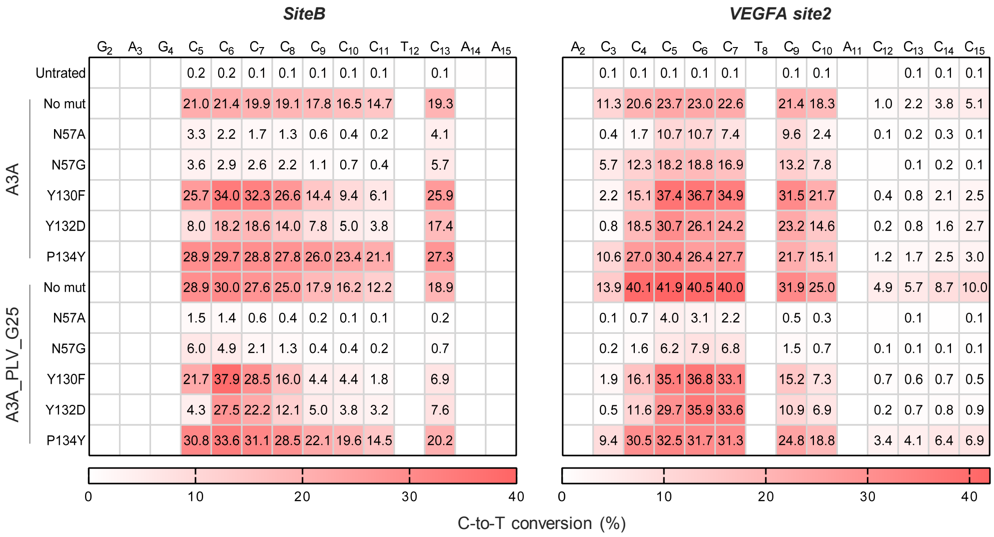

Figure S2. C-to-T conversions for point mutated A3A and A3A_PLV_G25. *SiteB* and *VEGFA site2* with multiple cytosines were tested. Editing efficiency shown represent the mean of three biologically independent replicates and those higher than 0.1% are labeled in cells.

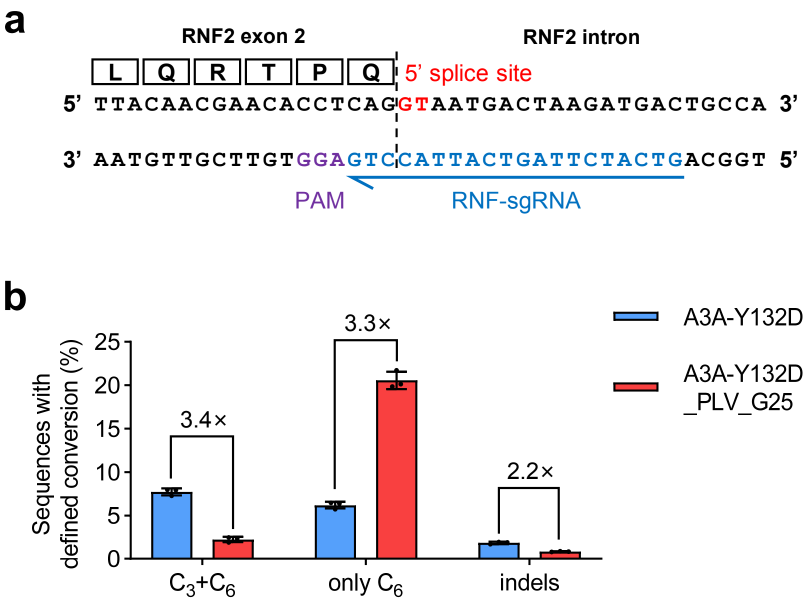

Figure S3. Comparison of base editing precision of A3A and PICBE in RNF2 locus. a) Schematic of RNF2 exon 2 and intron. The target sequences, 5' splice site, and PAM sequences are marked in blue, red, and purple, respectively. b) The proportions of edited cells with C_3+C_6-to-T_3+T_6, only C_6-to-T_6, and unwanted indels at RNF2 for A3A-Y132D and A3A-Y132D_PLV_G25.

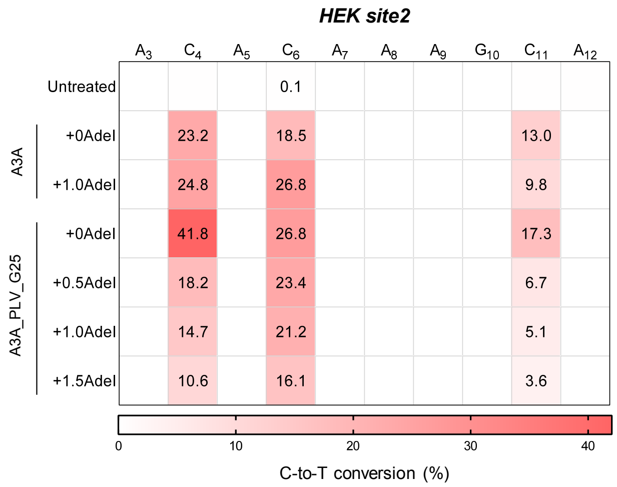

Figure S4. C-to-T conversions for A3A and A3A_PLV_G25 with AdeI at different doses. *HEK site2* was tested. Editing efficiency shown represent the mean of three biologically independent replicates and those higher than 0.1% are labeled in cells. The number in row labels means the concentration ratio of AdeI : A3A_PLV_G25.

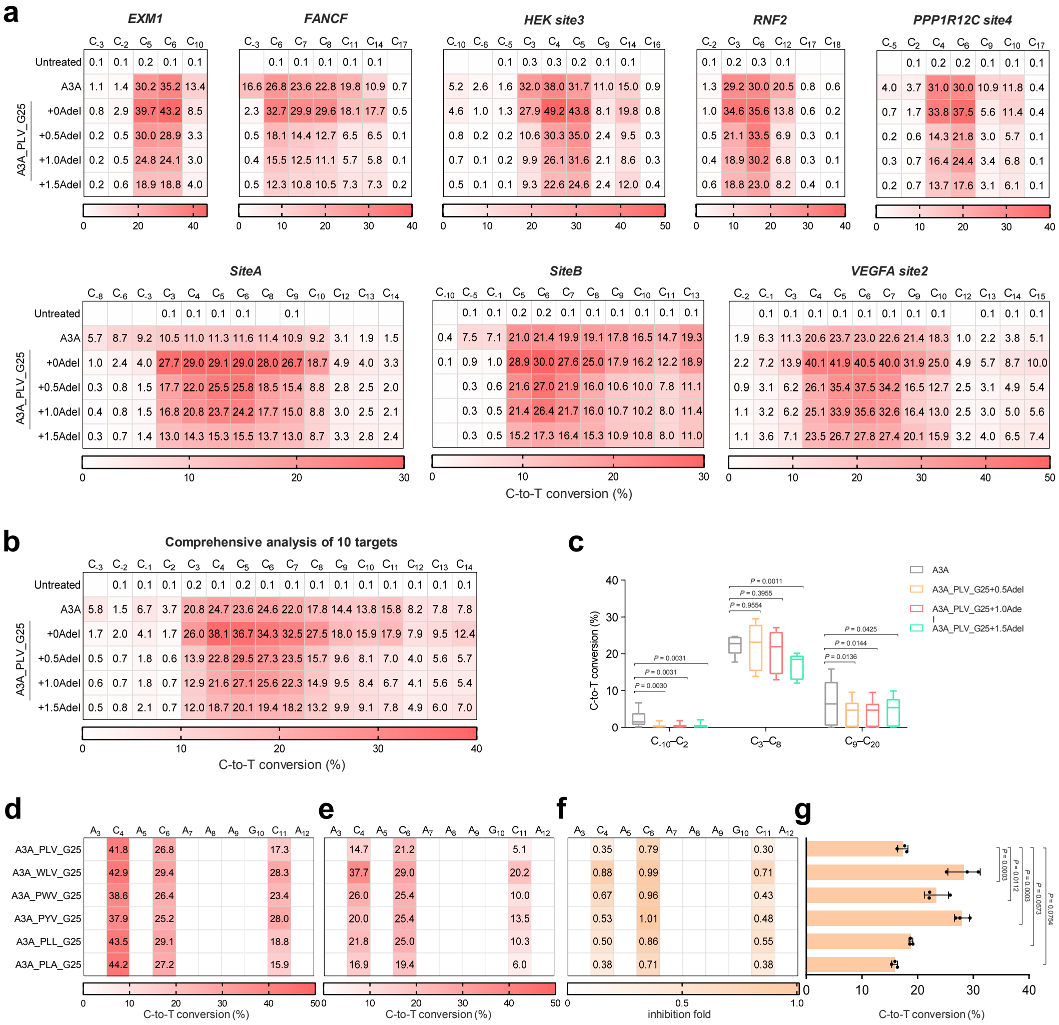

Figure S5. C-to-T conversions for A3A_PLV_G25 with AdeI at different doses. a) Eight endogenous loci were tested. b) Average C-to-T conversions for A3A and A3A_PLV_G25 with different doses of AdeI at nine endogenous loci shown in (a) and Figure S3. c) the editing efficiency of A3A_PLV_G25 with different doses of AdeI at PAM-distal (C_{-10}–C_2), PAM-proximal (C_9–C_{20}), and primary positions (C_3–C_8) compared to A3A. d,e) C-to-T conversions for A3A with different triplets insertion with (d) or without (e) 1.0AdeI at *HEK site2*. f) AdeI's inhibition rate for A3A with different triplets insertion calculated using the editing efficiency of (d)/that of (e). g) C-to-T conversions for A3A with different triplets insertion at the position C_{11} of *HEK site2*. Editing efficiency shown represent the mean of three biologically independent replicates and those higher than 0.1% are labeled in cells. The number in row labels means the concentration ratio of AdeI : A3A_PLV_G25.

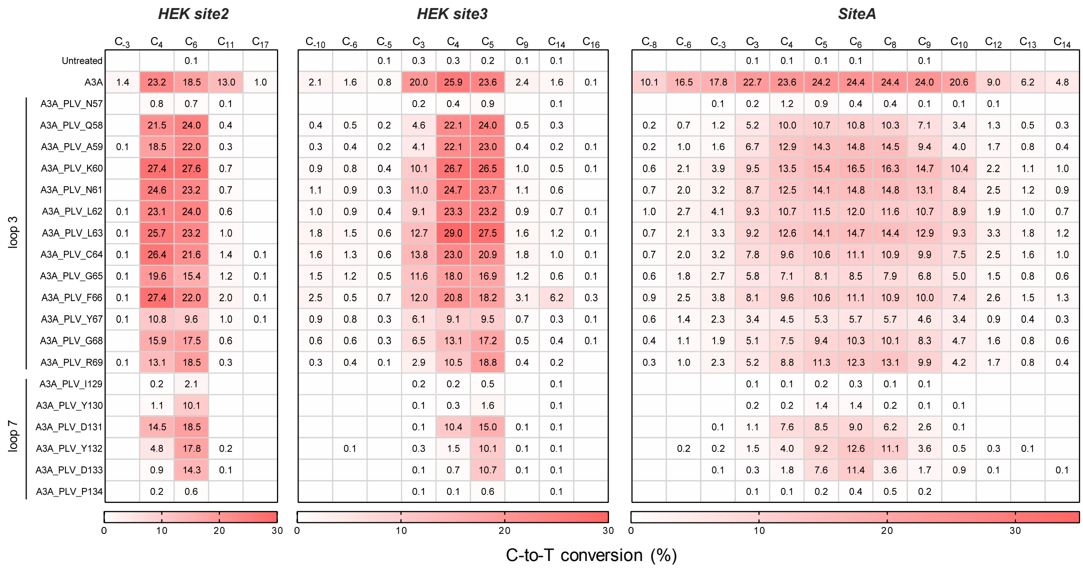

Figure S6. Test of PICBEs with PLV inserted in various regions. *HEK site2*, *HEK site3*, and *SiteA* were tested. Editing efficiency shown represent the mean of three biologically independent replicates and those higher than 0.1% are labeled in cells.

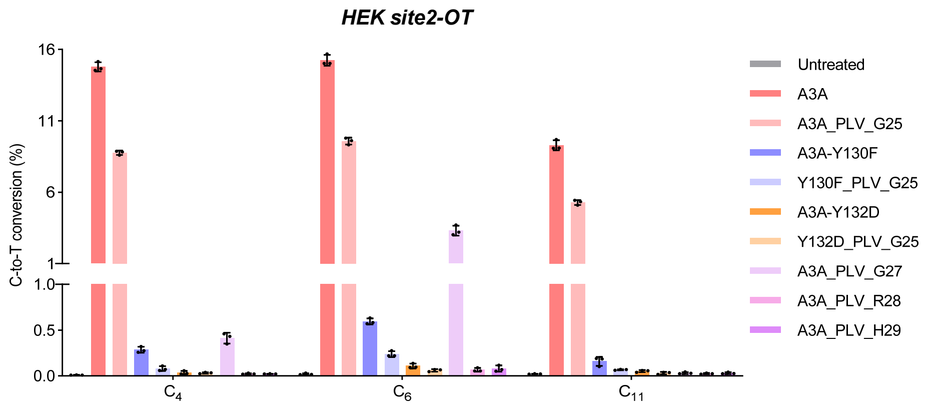

Figure S7. Comparison of sgRNA-dependent off-target effects of PICBEs. HEK site2-OT was tested. All data are shown as individual data points and means ±s.d. for *n* = 3 independent biological replicates.

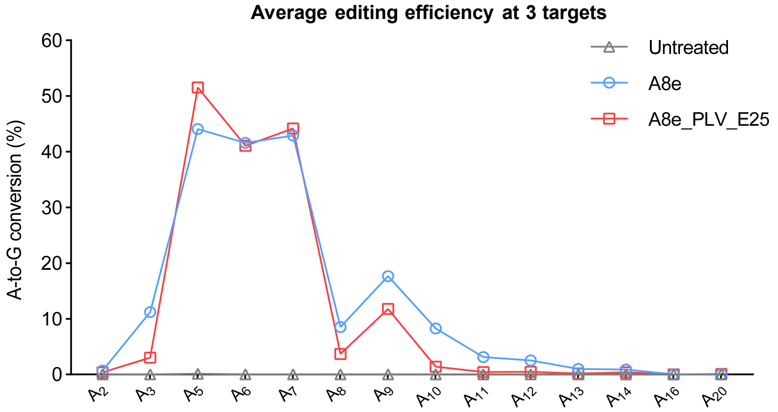

Figure S8. Comparison of the on-target activity of A8e and A8e_PLV_E25. Average on-target editing efficiency for A8e and A8e_PLV_E25 from three endogenous loci shown in Figure 5a.

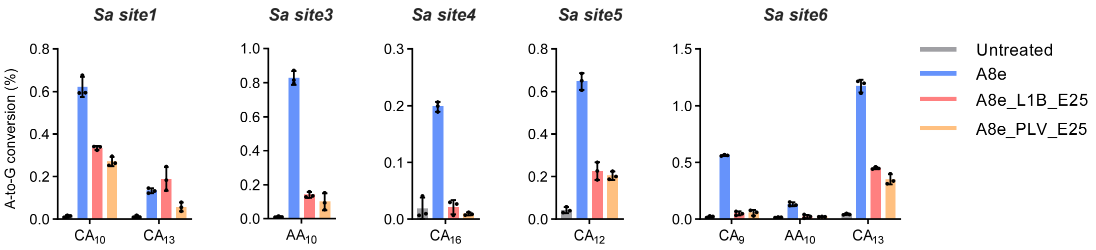

Figure S9. Comparison of the sgRNA-independent off-target effects of A8e and A8e_L1B/PLV_E25. The sgRNA-independent off-target editing frequency for A8e and A8e_L1B/PLV_E25 at five SaCas9 loci. All data are shown as individual data points and means ± s.d. for n = 3 independent biological replicates.

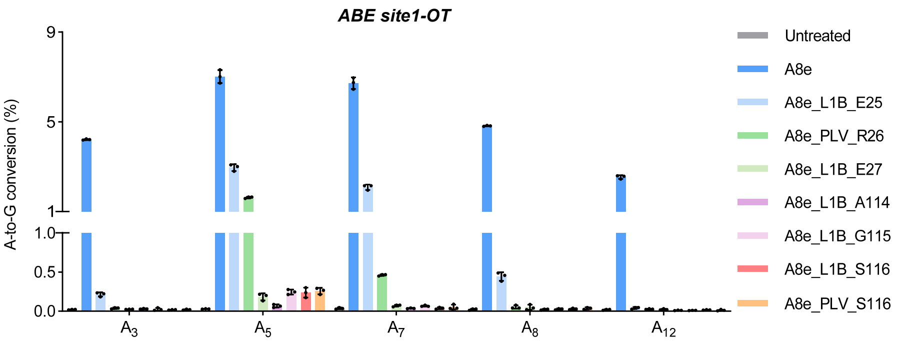

Figure S10. Comparison of sgRNA-dependent off-target effects of PIABEs. *ABE site1-OT* was tested. All data are shown as individual data points and means ± s.d. for n = 3 independent biological replicates.

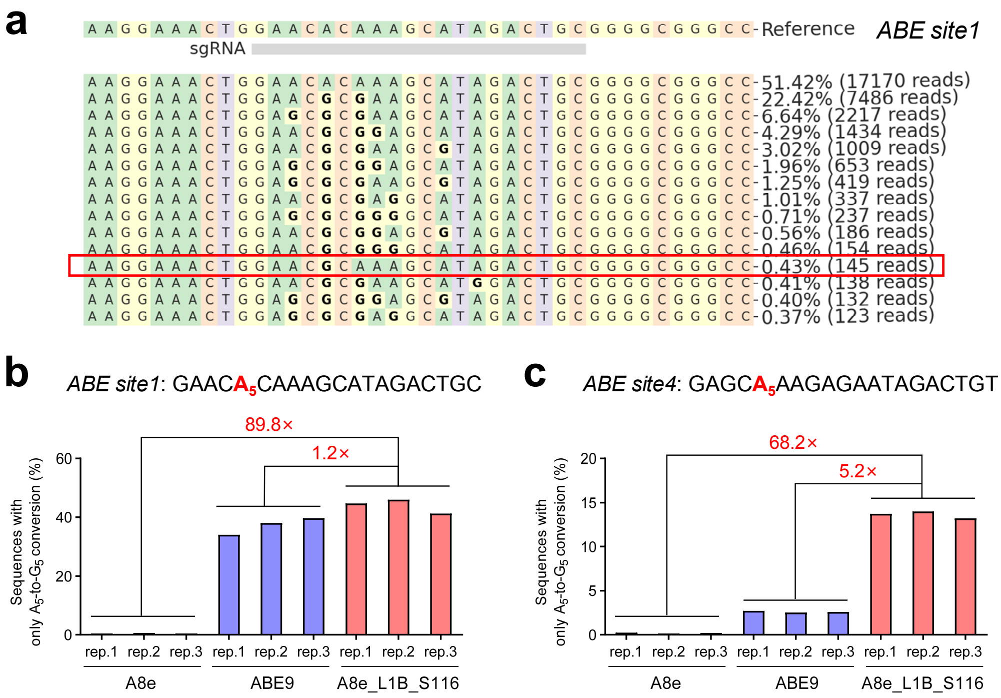

Figure S11. Comparison of base editing precision of A8e, ABE9, and PIABE. a) Allele frequencies of cells edited by A8e at *ABE site1*. The sequence boxed in red represent the edited cell with only A_5-to-G_5 conversion at *ABE site1*.b) The proportions of edited cells with only A_5-to-G_5 conversion at *ABE site1* for A8e, ABE9, and A8e_L1B_S116, respectively. c) The proportions of edited cells with only A_5-to-G_5 conversion at *ABE site4* for A8e, ABE9, and A8e_L1B_S116, respectively.

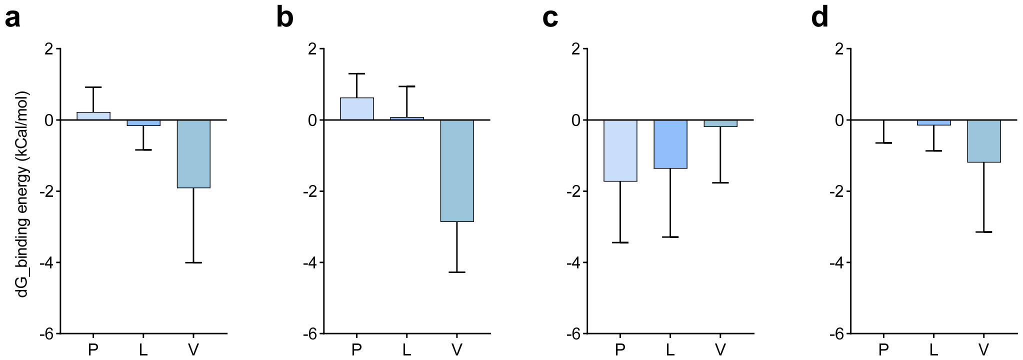

Figure S12. Binding energy for PLV triplet. a–d) Binding energy for PLV triplet in the complex of A8e_PLV_S116 with ssDNA containing (a) $C_{-1}A_0C_1$ (b) $T_{-1}A_0C_1$ (c) $A_{-1}A_0C_1$ (d) $G_{-1}A_0C_1$ motifs. Molecular dynamics simulations of 100 ns are performed and the binding energy are calculated from the simulation trajectory with a step_size of 1 ns.

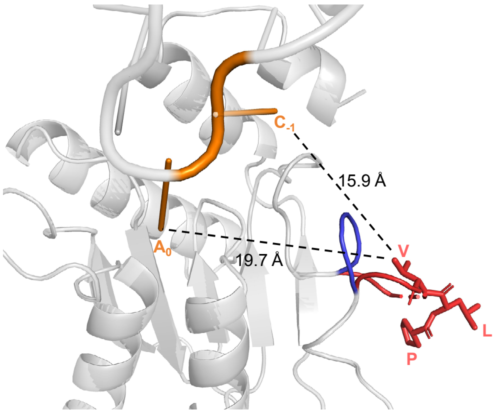

Figure S13. Structure comparison of A8e and A8e_PLV_S116. The structure of A8e and A8e_PLV_S116 with ssDNA. The same structures are shown in gray. The difference A8e and A8e_PLV_S116 are marked in blue and red, respectively. The PLV triplet in A8e_PLV_S116 is shown as sticks. The distance from V to the key bases C_{-1} and A_0 (orange) are calculated and displayed.

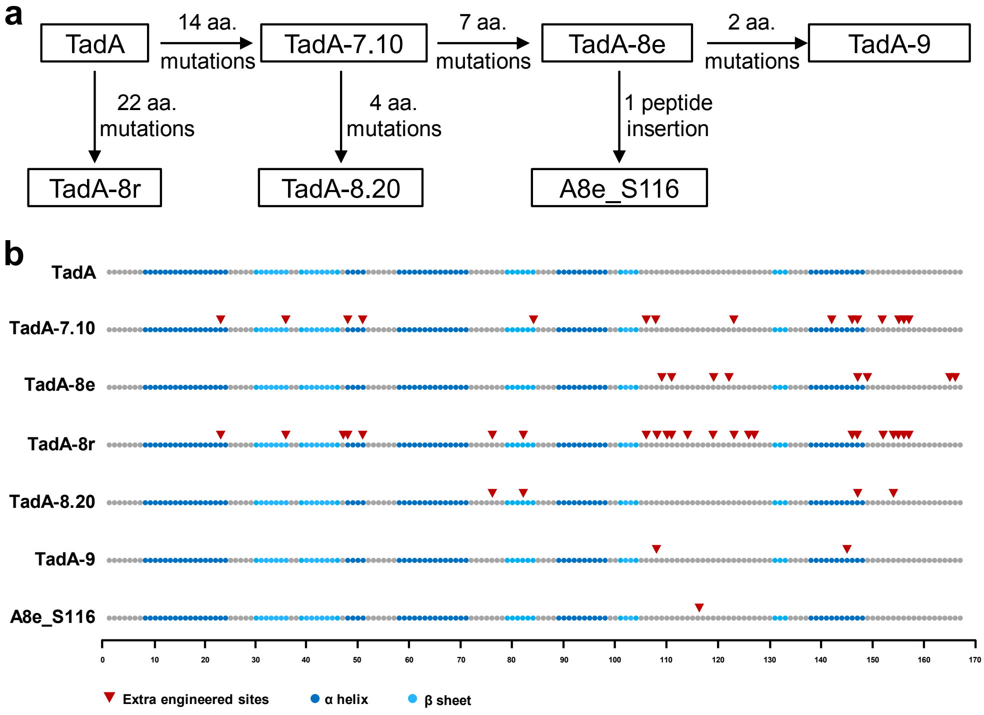

Figure S14. Comparison of TadA-based ABEs developed by different engineering methods. a) Engineering process of the TadA-based ABEs. b) Schematic of the mutation sites on the primary structure of TadA. The sites for point mutation or peptide insertion are marked in the red triangle. The secondary structure of TadA is highlighted with different colors (α-helix, dark blue; β-sheet, light blue). aa. means amino acid. A8e_S116 represents the PIABE, A8e_L1B/PLV_S116, developed in this study.

Supporting Tables

Table S1. Target sgRNA-protospacer sequences in this study.

Locus	Target protospacer sequence	PAM	Primer sequence for amplicon sequencing (5'→3')	
EXM1	GAGTCCGAGCAGAAGAAGAA	GGG	sgRNA-F	ATCAGGCTCTCAGCTCAGCC
			sgRNA-R	GCTTCGTGGCAATGCGCCAC
FANCF	GGAATCCCTTCTGCAGCACC	TGG	sgRNA-F	GGGTCCCAGGTGCTGACGTA
			sgRNA-R	GACGCTGGGAGATTGACATGC
HEK site2	GAACACAAAGCATAGACTGC	GGG	sgRNA-F	TGAATGGATTCCTTGGAAACAATGA
			sgRNA-R	CCAGCCCCATCTGTCAAACT
HEK site3	GGCCCAGACTGAGCACGTGA	TGG	sgRNA-F	ATGTGGGCTGCCTAGAAAGG
			sgRNA-R	GCGACGCCCTCTGGAGGAAG
HEK site4	GGCACTGCGGCTGGAGGTGG	GGG	sgRNA-F	CAAGATGGCTGACAAAGGCCG
			sgRNA-R	CTCCTTTCAACCCGAACGGAG
RNF2	GTCATCTTAGTCATTACCTG	AGG	sgRNA-F	ATGTCTCAGGCTGTGCAGAC
			sgRNA-R	GCCAACATACAGAAGTCAGGAA
SiteA	TGCCCCTCCCTCCCTGGCCC	AGG	sgRNA-F	ATCAGGCTCTCAGCTCAGCC
			sgRNA-R	GCTTCGTGGCAATGCGCCAC
SiteB	AGAGCCCCCCCTCAAAGAGA	GGG	sgRNA-F	GAGTCCTTGGCAAGCTGCTG
			sgRNA-R	CCAGGGCAGTAGTAGGAGGAC
VEGFA site2	GACCCCCTCCACCCCGCCTC	CGG	sgRNA-F	GCGCTGACGGACAGACAGACAG
			sgRNA-R	GAAGCGAGAACAGCCCAGAAGTTGG
CBE site10	GACAAACCAGAAGCCGCTCC	TGG	sgRNA-F	GCCCTCTTTTTATTGGAACTGTG
			sgRNA-R	CCTATCACAATCACAACTGCAA
CBE site11	GTTCACACCCATGACGAACA	TGG	sgRNA-F	CTGACTCAGCCCTGCAAAGG
			sgRNA-R	CATAGGGTGCCAGGCTGGCC
WFS	CAGCAGTATGGTGCGCTGTG	CGG	sgRNA-F	GTGCTGTTCTGCTGGTTCTATGTG
			sgRNA-R	GTTGATGGCAGACTCGGCGC
PPP1R12C site2	GTCCGACTCGGCCAGGTCCA	GGG	sgRNA-F	GGGCCAACATCGCCGCCGTC
			sgRNA-R	AAGACAATCCTAGGAAGCAGGGT
PPP1R12C site3	GACCCTCAGCCGTGCTGCTC	GGG	sgRNA-F	GGATCAGCCAGGAACGAAGAC
			sgRNA-R	GCGTGTCCTGCAGGGGGTGC
PPP1R12C site4	GCTCTCAGCCTGGAGACCAC	GGG	sgRNA-F	CAACATCGCCGCCGTCAACAG
			sgRNA-R	AAGACAATCCTAGGAAGCAGGG
PPP1R12C site5	GCTGACTCAGAGACCCTGAG	TGG	sgRNA-F	GGAAAGGCCTGTGATCTCCG
			sgRNA-R	GACTCACACCCCATGACCTG
PPP1R12C site6	GGGGCTCAACATCGGAAGAG	GGG	sgRNA-F	GCCAGGCAGATAGACCAGACTG
			sgRNA-R	CCCCTTCGACCTACTCTCTTC
PPP1R12C site7	GGCACTCGGGGGCGAGAGGA	GGG	sgRNA-F	GCTCAGTCTGGTCTATCTGCC
			sgRNA-R	GGAGAGAATGCAGGTCAGAGAA
EMX1 site2	GTATTCACCTGAAAGTGTGC	AGG	sgRNA-F	GAGAGTGGGAACAGCCCACATG
			sgRNA-R	GAATGAACGAGGTCTGGATCCC

CTNNB1 site1	CTGGACTCTGGAATCCATTC	TGG	sgRNA-F	TGGACATGGCCATGGAACCA
			sgRNA-R	CCAGCTACTTGTTCTTGAGTGAAG
ABE Site1	GAACACAAAGCATAGACTGC	GGG	sgRNA-F	TGAATGGATTCCTTGGAAACAATGA
			sgRNA-R	CCAGCCCCATCTGTCAAACT
ABE Site2	GAGTATGAGGCATAGACTGC	AGG	sgRNA-F	TAGTTTCCCTGAGATACAGTCACG
			sgRNA-R	TGAAATGCTGTGCGTGTCTAAC
ABE Site3	GTCAAGAAAGCAGAGACTGC	CGG	sgRNA-F	TGAAGAAGGATGCAAGTTTGTC
			sgRNA-R	ATTGAGGCTCAGAGGAGATGTG
ABE Site4	GAGCAAAGAGAATAGACTGT	AGG	sgRNA-F	TCGATCTCCTGACCTCGTGATC
			sgRNA-R	ACAGAGAGTTACTGCTCAGACA
ABE Site5	GATGAGATAATGATGAGTCA	GGG	sgRNA-F	CCATCTTACCCTCCTCAGAAGT
			sgRNA-R	CCGCGCCTGGTCACATTGAC
ABE Site6	GGATTGACCCAGGCCAGGGC	TGG	sgRNA-F	ATGTGGGCTGCCTAGAAAGG
			sgRNA-R	GCGACGCCCTCTGGAGGAAG
ABE Site7	GAATACTAAGCATAGACTCC	AGG	sgRNA-F	GATGCCCTCCATCTTCTCCG
			sgRNA-R	AGGTTTGCATAGACCTGCCC
ABE Site8	GTAAACAAAGCATAGACTGA	GGG	sgRNA-F	GCCCCAAATTATTCACTACCCC
			sgRNA-R	GAATAGTTTAAGCAAGGCTGCTAG
ABE Site9	GAAGACCAAGGATAGACTGC	TGG	sgRNA-F	CCTACACGCCTGACAAAGCT
			sgRNA-R	CGGACAGTGAAGAGAGTATTTG
ABE Site10	GAACATAAAGAATAGAATGA	TGG	sgRNA-F	CACAGTGGAGTATCCATCACTTC
			sgRNA-R	AAGAATGGAATCATGTCATTTGCA
ABE Site11	GGACAGGCAGCATAGACTGT	GGG	sgRNA-F	CCCTAAACCACCTGCAGAGG
			sgRNA-R	CAAAGACAAGAACTCGAACTCC
ABE Site12	GTAGAAAAAGTATAGACTGC	AGG	sgRNA-F	TGGTGATTATGGTTACACAGCG
			sgRNA-R	CTCCTTAGTACAAAGGCAATTAAA
ABE Site13	GAAGATAGAGAATAGACTGC	TGG	sgRNA-F	GATCTATCAAACACTGGGTCATAC
			sgRNA-R	TCACTTCAGCCCAGGAGTAT
ABE Site14	GGCTAAAGACCATAGACTGT	GGG	sgRNA-F	AACCTGTGTGACACTTGGCA
			sgRNA-R	CACAACTATTTCATGGCTGAGT
ABE Site15	GTCTAGAAAGCTTAGACTGC	TGG	sgRNA-F	TCTGCATCAGAAAGGATCTCTC
			sgRNA-R	GCACTCAGCTAGACTTAACTCCC
ABE Site16	GGGAATAAATCATAGAATCC	TGG	sgRNA-F	GGTCTGTGCCAGTCCCCCAA
			sgRNA-R	GGGAGGTGGAGAGAGGATGT
ABE Site17	GACAAAGAGGAAGAGAGACG	GGG	sgRNA-F	CGCGGGCTGAAGTAGATCAA
			sgRNA-R	ATCCCCCACCTTCCCTCCTC

Table S2. Off-target sgRNA-protospacer sequences in this study.

Locus	Target protospacer sequence	PAM	Primer sequence for amplicon sequencing (5'→3')	
HEK Site2-OT	GAACACAATGCATAGATTGC	CGG	sgRNA-F	AAGCCAGAGTTAAGAAGTTGG
			sgRNA-R	ACATATTTAATGCTCCCACACC
Sa site1	GTGGTAGACAGCATGTGTCCTA	AAGGGT	sgRNA-F	TGCAGTCTCCTGCTTCTCTG
			sgRNA-R	GGCAACAAGCTTCTGTACCA
Sa site3	GTGTCAGGTAATGTGCTAAACA	GAGAGT	sgRNA-F	TTGCTCCAGATTTCCCTTCA
			sgRNA-R	GTGCTGTGGCATCCAGAGACAT
Sa site4	GGTGGAGGAGGGTGCATGGGGT	CAGAAT	sgRNA-F	TCCTGAGGTCTAGGAACCCG
			sgRNA-R	TCATAGAATCCTGGACAAGG
Sa site5	TCTGCTTCTCCAGCCCTGGC	CTGGGT	sgRNA-F	ATGTGGGCTGCCTAGAAAGG
			sgRNA-R	GCGACGCCCTCTGGAGGAAG
Sa site6	GATGTTCCAATCAGTACGCA	GAGAGT	sgRNA-F	CTAACACGGCGGGGTCCCAGGTGCTGAC
			sgRNA-R	CTTACCGGTAGCATTGCAGAGAGGCGTATC

Table S3. Inserted peptide sequences for PIBEs.

Construct name	Inserted peptide	Amino acid sequences	Inserted positions in A3A or A8e
A3A_L1B_G25	L1B	DPLVLRRRQ	SNFNN**G$_{25}$I$_{26}$**GRHKT
A3A_PLV_G25	PLV	PLV	SNFNN**G$_{25}$I$_{26}$**GRHKT
A3A_L1G_G25	L1G	EPWVRGRHE	SNFNN**G$_{25}$I$_{26}$**GRHKT
A3A_PWV_G25	PWV	PWV	SNFNN**G$_{25}$I$_{26}$**GRHKT
A3A_PLV_I26	PLV	PLV	NFNNG**I$_{26}$G$_{27}$**RHKTY
A3A_PLV_G27	PLV	PLV	FNNGI**G$_{27}$R$_{28}$**HKTYL
A3A_PLV_R28	PLV	PLV	NNGIG**R$_{28}$H$_{29}$**KTYLC
A3A_PLV_H29	PLV	PLV	NGIGR**H$_{29}$K$_{30}$**TYLCY
A3A_PLV_K30	PLV	PLV	GIGRH**K$_{30}$T$_{31}$**YLCYE
A3A_PLV_N57	PLV	PLV	RGFLH**N$_{57}$Q$_{58}$**AKNLL
A3A_PLV_Q58	PLV	PLV	GFLHN**Q$_{58}$A$_{59}$**KNLLC
A3A_PLV_A59	PLV	PLV	FLHNQ**A$_{59}$K$_{60}$**NLLCG
A3A_PLV_K60	PLV	PLV	LHNQA**K$_{60}$N$_{61}$**LLCGF
A3A_PLV_N61	PLV	PLV	HNQAK**N$_{61}$L$_{62}$**LCGFY
A3A_PLV_L62	PLV	PLV	NQAKN**L$_{62}$L$_{63}$**CGFYG
A3A_PLV_L63	PLV	PLV	QAKNL**L$_{63}$C$_{64}$**GFYGR
A3A_PLV_C64	PLV	PLV	AKNLL**C$_{64}$G$_{65}$**FYGRH
A3A_PLV_G65	PLV	PLV	KNLLC**G$_{65}$F$_{66}$**YGRHA
A3A_PLV_F66	PLV	PLV	NLLCG**F$_{66}$Y$_{67}$**GRHAE
A3A_PLV_Y67	PLV	PLV	LLCGF**Y$_{67}$G$_{68}$**RHAEL
A3A_PLV_G68	PLV	PLV	LCGFY**G$_{68}$R$_{69}$**HAELR
A3A_PLV_R69	PLV	PLV	CGFYG**R$_{69}$H$_{70}$**AELRF
A3A_PLV_I129	PLV	PLV	IFAAR**I$_{129}$Y$_{130}$**DYDPL
A3A_PLV_Y130	PLV	PLV	FAARI**Y$_{130}$D$_{131}$**YDPLY
A3A_PLV_D131	PLV	PLV	AARIY**D$_{131}$Y$_{132}$**DPLYK
A3A_PLV_Y132	PLV	PLV	ARIYD**Y$_{132}$D$_{133}$**PLYKE
A3A_PLV_D133	PLV	PLV	RIYDY**D$_{133}$P$_{134}$**LYKEA
A3A_PLV_P134	PLV	PLV	IYDYD**P$_{134}$L$_{135}$**YKEAL
A8e_L1B_E27	L1B	DPLVLRRRQ	ARDER**E$_{27}$V$_{28}$**PVGAV
A8e_L1B_N37	L1B	DPLVLRRRQ	AVLVL**N$_{37}$N$_{38}$**RVIGE
A8e_L1B_I49	L1B	DPLVLRRRQ	GWNRA**I$_{49}$G$_{50}$**LHDPT
A8e_L1B_D53	L1B	DPLVLRRRQ	AIGLH**D$_{53}$P$_{54}$**TAHAE
A8e_L1B_R74	L1B	DPLVLRRRQ	VMQNY**R$_{74}$L$_{75}$**IDATL
A8e_L1B_I99	L1B	DPLVLRRRQ	MIHSR**I$_{99}$G$_{100}$**RVVFG
A8e_L1B_S109	L1B	DPLVLRRRQ	FGVRN**S$_{109}$K$_{110}$**RGAAG
A8e_L1B_A114	L1B	DPLVLRRRQ	SKRGA**A$_{114}$G$_{115}$**SLMNV
A8e_L1B_P124	L1B	DPLVLRRRQ	NVLNY**P$_{124}$G$_{125}$**MNHRV
A8e_L1B_G135	L1B	DPLVLRRRQ	VEITE**G$_{135}$I$_{136}$**LADEC
A8e_L1B_E25	L1B	DPLVLRRRQ	KRARD**E$_{25}$R$_{26}$**EVPVG
A8e_PLV_E25	PLV	PLV	KRARD**E$_{25}$R$_{26}$**EVPVG

A8e_L1B_R26	L1B	DPLVLRRRQ	RARDE**R**$_{26}$**E**$_{27}$VPVGA
A8e_PLV_R26	PLV	PLV	RARDE**R**$_{26}$**E**$_{27}$VPVGA
A8e_L1B_V28	L1B	DPLVLRRRQ	RDERE**V**$_{28}$**P**$_{29}$VGAVL
A8e_PLV_V28	PLV	PLV	RDERE**V**$_{28}$**P**$_{29}$VGAVL
A8e_L1B_P29	L1B	DPLVLRRRQ	DEREV**P**$_{29}$**V**$_{30}$GAVLV
A8e_PLV_P29	PLV	PLV	DEREV**P**$_{29}$**V**$_{30}$GAVLV
A8e_PLV_E27	PLV	PLV	ARDER**E**$_{27}$**V**$_{28}$PVGAV
A8e_L1B_G112	L1B	DPLVLRRRQ	RNSKR**G**$_{112}$**A**$_{113}$AGSLM
A8e_PLV_G112	PLV	PLV	RNSKR**G**$_{112}$**A**$_{113}$AGSLM
A8e_L1B_A113	L1B	DPLVLRRRQ	NSKRG**A**$_{113}$**A**$_{114}$GSLMN
A8e_PLV_A113	PLV	PLV	NSKRG**A**$_{113}$**A**$_{114}$GSLMN
A8e_L1B_G115	L1B	DPLVLRRRQ	KRGAA**G**$_{115}$**S**$_{116}$LMNVL
A8e_PLV_G115	PLV	PLV	KRGAA**G**$_{115}$**S**$_{116}$LMNVL
A8e_L1B_S116	L1B	DPLVLRRRQ	RGAAG**S**$_{116}$**L**$_{117}$MNVLN
A8e_PLV_S116	PLV	PLV	RGAAG**S**$_{116}$**L**$_{117}$MNVLN
A8e_PLV_A114	PLV	PLV	SKRGA**A**$_{114}$**G**$_{115}$SLMNV

Supporting Sequences

Plasmids (functional amino acid sequences shown only) for HEK293T in this study.

pCMV-A3A (A3A-linker-nCas9-linker-UGI-NLS)

MEASPASGPRHLMDPHIFTSNFNNGIGRHKTYLCYEVERLDNGTSVKMDQHRGFLHNQAKNLLCGFYGR
HAELRFLDLVPSLQLDAQIYRVTWFISWSPCFSWGCAGEVRAFLQENTHVRLRIFAARIYDYDPLYKEA
LQMLRDAGAQVSIMTYDEFKHCWDTFVDHQGCPFQPWDGLDEHSQALSGRLRAILQNQGNSGSETPGTS
ESATPESDKKYSIGLAIGTNSVGWAVITDEYKVPSKKFKVLGNTDRHSIKKNLIGALLFDSGETAEATR
LKRTARRRYTRRKNRICYLQEIFSNEMAKVDDSFFHRLEESFLVEEDKKHERHPIFGNIVDEVAYHEKY
PTIYHLRKKLVDSTDKADLRLIYLALAHMIKFRGHFLIEGDLNPDNSDVDKLFIQLVQTYNQLFEENPI
NASGVDAKAILSARLSKSRRLENLIAQLPGEKKNGLFGNLIALSLGLTPNFKSNFDLAEDAKLQLSKDT
YDDDLDNLLAQIGDQYADLFLAAKNLSDAILLSDILRVNTEITKAPLSASMIKRYDEHHQDLTLLKALV
RQQLPEKYKEIFFDQSKNGYAGYIDGGASQEEFYKFIKPILEKMDGTEELLVKLNREDLLRKQRTFDNG
SIPHQIHLGELHAILRRQEDFYPFLKDNREKIEKILTFRIPYYVGPLARGNSRFAWMTRKSEETITPWN
FEEVVDKGASAQSFIERMTNFDKNLPNEKVLPKHSLLYEYFTVYNELTKVKYVTEGMRKPAFLSGEQKK
AIVDLLFKTNRKVTVKQLKEDYFKKIECFDSVEISGVEDRFNASLGTYHDLLKIIKDKDFLDNEENEDI
LEDIVLTLTLFEDREMIEERLKTYAHLFDDKVMKQLKRRRYTGWGRLSRKLINGIRDKQSGKTILDFLK
SDGFANRNFMQLIHDDSLTFKEDIQKAQVSGQGDSLHEHIANLAGSPAIKKGILQTVKVVDELVKVMGR
HKPENIVIEMARENQTTQKGQKNSRERMKRIEEGIKELGSQILKEHPVENTQLQNEKLYLYYLQNGRDM
YVDQELDINRLSDYDVDHIVPQSFLKDDSIDNKVLTRSDKNRGKSDNVPSEEVVKKMKNYWRQLLNAKL
ITQRKFDNLTKAERGGLSELDKAGFIKRQLVETRQITKHVAQILDSRMNTKYDENDKLIREVKVITLKS
KLVSDFRKDFQFYKVREINNYHHAHDAYLNAVVGTALIKKYPKLESEFVYGDYKVYDVRKMIAKSEQEI
GKATAKYFFYSNIMNFFKTEITLANGEIRKRPLIETNGETGEIVWDKGRDFATVRKVLSMPQVNIVKKT
EVQTGGFSKESILPKRNSDKLIARKKDWDPKKYGGFDSPTVAYSVLVVAKVEKGKSKKLKSVKELLGIT
IMERSSFEKNPIDFLEAKGYKEVKKDLIIKLPKYSLFELENGRKRMLASAGELQKGNELALPSKYVNFL
YLASHYEKLKGSPEDNEQKQLFVEQHKHYLDEIIEQISEFSKRVILADANLDKVLSAYNKHRDKPIREQ
AENIIHLFTLTNLGAPAAFKYFDTTIDRKRYTSTKEVLDATLIHQSITGLYETRIDLSQLGGDSGGSTN
LSDIIEKETGKQLVIQESILMLPEEVEEVIGNKPESDILVHTAYDESTDENVMLLTSDAPEYKPWALVI
QDSNGENKIKMLSGGSPKKKRKV*

pCMV-A3A_PLV_G25 (A3A_PLV_G25-linker-nCas9-linker-UGI-NLS)

MEASPASGPRHLMDPHIFTSNFNNGPLVIGRHKTYLCYEVERLDNGTSVKMDQHRGFLHNQAKNLLCGF
YGRHAELRFLDLVPSLQLDAQIYRVTWFISWSPCFSWGCAGEVRAFLQENTHVRLRIFAARIYDYDPLY
KEALQMLRDAGAQVSIMTYDEFKHCWDTFVDHQGCPFQPWDGLDEHSQALSGRLRAILQNQGNSGSETP
GTSESATPESDKKYSIGLAIGTNSVGWAVITDEYKVPSKKFKVLGNTDRHSIKKNLIGALLFDSGETAE
ATRLKRTARRRYTRRKNRICYLQEIFSNEMAKVDDSFFHRLEESFLVEEDKKHERHPIFGNIVDEVAYH

EKYPTIYHLRKKLVDSTDKADLRLIYLALAHMIKFRGHFLIEGDLNPDNSDVDKLFIQLVQTYNQLFEE
NPINASGVDAKAILSARLSKSRRLENLIAQLPGEKKNGLFGNLIALSLGLTPNFKSNFDLAEDAKLQLS
KDTYDDDLDNLLAQIGDQYADLFLAAKNLSDAILLSDILRVNTEITKAPLSASMIKRYDEHHQDLTLLK
ALVRQQLPEKYKEIFFDQSKNGYAGYIDGGASQEEFYKFIKPILEKMDGTEELLVKLNREDLLRKQRTF
DNGSIPHQIHLGELHAILRRQEDFYPFLKDNREKIEKILTFRIPYYVGPLARGNSRFAWMTRKSEETIT
PWNFEEVVDKGASAQSFIERMTNFDKNLPNEKVLPKHSLLYEYFTVYNELTKVKYVTEGMRKPAFLSGE
QKKAIVDLLFKTNRKVTVKQLKEDYFKKIECFDSVEISGVEDRFNASLGTYHDLLKIIKDKDFLDNEEN
EDILEDIVLTLTLFEDREMIEERLKTYAHLFDDKVMKQLKRRRYTGWGRLSRKLINGIRDKQSGKTILD
FLKSDGFANRNFMQLIHDDSLTFKEDIQKAQVSGQGDSLHEHIANLAGSPAIKKGILQTVKVVDELVKV
MGRHKPENIVIEMARENQTTQKGQKNSRERMKRIEEGIKELGSQILKEHPVENTQLQNEKLYLYYLQNG
RDMYVDQELDINRLSDYDVDHIVPQSFLKDDSIDNKVLTRSDKNRGKSDNVPSEEVVKKMKNYWRQLLN
AKLITQRKFDNLTKAERGGLSELDKAGFIKRQLVETRQITKHVAQILDSRMNTKYDENDKLIREVKVIT
LKSKLVSDFRKDFQFYKVREINNYHHAHDAYLNAVVGTALIKKYPKLESEFVYGDYKVYDVRKMIAKSE
QEIGKATAKYFFYSNIMNFFKTEITLANGEIRKRPLIETNGETGEIVWDKGRDFATVRKVLSMPQVNIV
KKTEVQTGGFSKESILPKRNSDKLIARKKDWDPKKYGGFDSPTVAYSVLVVAKVEKGKSKKLKSVKELL
GITIMERSSFEKNPIDFLEAKGYKEVKKDLIIKLPKYSLFELENGRKRMLASAGELQKGNELALPSKYV
NFLYLASHYEKLKGSPEDNEQKQLFVEQHKHYLDEIIEQISEFSKRVILADANLDKVLSAYNKHRDKPI
REQAENIIHLFTLTNLGAPAAFKYFDTTIDRKRYTSTKEVLDATLIHQSITGLYETRIDLSQLGGDSGG
STNLSDIIEKETGKQLVIQESILMLPEEVEEVIGNKPESDILVHTAYDESTDENVMLLTSDAPEYKPWA
LVIQDSNGENKIKMLSGGSPKKKRKV*

pCMV-A8e (A8e-linker-nCas9-linker-UGI-NLS)

MSEVEFSHEYWMRHALTLAKRARDEREVPVGAVLVLNNRVIGEGWLRAIGLHDPTAHAEIMALRQGGLV
MQNYRLIDATLYVTFEPCVMCAGAMIHSRIGRVVFGVRNSKRGAAGSLMNVLNYPGMNHRVEITEGILA
DECAALLCDFYRMPRQVFNAQKKAQSSINSGGSSGGSSGSETPGTSESATPESSGGSSGGSDKKYSIGL
AIGTNSVGWAVITDEYKVPSKKFKVLGNTDRHSIKKNLIGALLFDSGETAEATRLKRTARRRYTRRKNR
ICYLQEIFSNEMAKVDDSFFHRLEESFLVEEDKKHERHPIFGNIVDEVAYHEKYPTIYHLRKKLVDSTD
KADLRLIYLALAHMIKFRGHFLIEGDLNPDNSDVDKLFIQLVQTYNQLFEENPINASGVDAKAILSARL
SKSRRLENLIAQLPGEKKNGLFGNLIALSLGLTPNFKSNFDLAEDAKLQLSKDTYDDDLDNLLAQIGDQ
YADLFLAAKNLSDAILLSDILRVNTEITKAPLSASMIKRYDEHHQDLTLLKALVRQQLPEKYKEIFFDQ
SKNGYAGYIDGGASQEEFYKFIKPILEKMDGTEELLVKLNREDLLRKQRTFDNGSIPHQIHLGELHAIL
RRQEDFYPFLKDNREKIEKILTFRIPYYVGPLARGNSRFAWMTRKSEETITPWNFEEVVDKGASAQSFI
ERMTNFDKNLPNEKVLPKHSLLYEYFTVYNELTKVKYVTEGMRKPAFLSGEQKKAIVDLLFKTNRKVTV
KQLKEDYFKKIECFDSVEISGVEDRFNASLGTYHDLLKIIKDKDFLDNEENEDILEDIVLTLTLFEDRE
MIEERLKTYAHLFDDKVMKQLKRRRYTGWGRLSRKLINGIRDKQSGKTILDFLKSDGFANRNFMQLIHD

DSLTFKEDIQKAQVSGQGDSLHEHIANLAGSPAIKKGILQTVKVVDELVKVMGRHKPENIVIEMARENQ

TTQKGQKNSRERMKRIEEGIKELGSQILKEHPVENTQLQNEKLYLYYLQNGRDMYVDQELDINRLSDYD

VDHIVPQSFLKDDSIDNKVLTRSDKNRGKSDNVPSEEVVKKMKNYWRQLLNAKLITQRKFDNLTKAERG

GLSELDKAGFIKRQLVETRQITKHVAQILDSRMNTKYDENDKLIREVKVITLKSKLVSDFRKDFQFYKV

REINNYHHAHDAYLNAVVGTALIKKYPKLESEFVYGDYKVYDVRKMIAKSEQEIGKATAKYFFYSNIMN

FFKTEITLANGEIRKRPLIETNGETGEIVWDKGRDFATVRKVLSMPQVNIVKKTEVQTGGFSKESILPK

RNSDKLIARKKDWDPKKYGGFDSPTVAYSVLVVAKVEKGKSKKLKSVKELLGITIMERSSFEKNPIDFL

EAKGYKEVKKDLIIKLPKYSLFELENGRKRMLASAGELQKGNELALPSKYVNFLYLASHYEKLKGSPED

NEQKQLFVEQHKHYLDEIIEQISEFSKRVILADANLDKVLSAYNKHRDKPIREQAENIIHLFTLTNLGA

PAAFKYFDTTIDRKRYTSTKEVLDATLIHQSITGLYETRIDLSQLGGDSGGSTNLSDIIEKETGKQLVI

QESILMLPEEVEEVIGNKPESDILVHTAYDESTDENVMLLTSDAPEYKPWALVIQDSNGENKIKMLSGG

SPKKKRKV*

pCMV-A8e_PLV_S116 (A8e_PLV_S116-linker-nCas9-linker-UGI-NLS)

MSEVEFSHEYWMRHALTLAKRARDEREVPVGAVLVLNNRVIGEGWLRAIGLHDPTAHAEIMALRQGGLV

MQNYRLIDATLYVTFEPCVMCAGAMIHSRIGRVVFGVRNSKRGAAGSPLVLMNVLNYPGMNHRVEITEG

ILADECAALLCDFYRMPRQVFNAQKKAQSSINSGGSSGGSSGSETPGTSESATPESSGGSSGGSDKKYS

IGLAIGTNSVGWAVITDEYKVPSKKFKVLGNTDRHSIKKNLIGALLFDSGETAEATRLKRTARRRYTRR

KNRICYLQEIFSNEMAKVDDSFFHRLEESFLVEEDKKHERHPIFGNIVDEVAYHEKYPTIYHLRKKLVD

STDKADLRLIYLALAHMIKFRGHFLIEGDLNPDNSDVDKLFIQLVQTYNQLFEENPINASGVDAKAILS

ARLSKSRRLENLIAQLPGEKKNGLFGNLIALSLGLTPNFKSNFDLAEDAKLQLSKDTYDDDLDNLLAQI

GDQYADLFLAAKNLSDAILLSDILRVNTEITKAPLSASMIKRYDEHHQDLTLLKALVRQQLPEKYKEIF

FDQSKNGYAGYIDGGASQEEFYKFIKPILEKMDGTEELLVKLNREDLLRKQRTFDNGSIPHQIHLGELH

AILRRQEDFYPFLKDNREKIEKILTFRIPYYVGPLARGNSRFAWMTRKSEETITPWNFEEVVDKGASAQ

SFIERMTNFDKNLPNEKVLPKHSLLYEYFTVYNELTKVKYVTEGMRKPAFLSGEQKKAIVDLLFKTNRK

VTVKQLKEDYFKKIECFDSVEISGVEDRFNASLGTYHDLLKIIKDKDFLDNEENEDILEDIVLTLTLFE

DREMIEERLKTYAHLFDDKVMKQLKRRRYTGWGRLSRKLINGIRDKQSGKTILDFLKSDGFANRNFMQL

IHDDSLTFKEDIQKAQVSGQGDSLHEHIANLAGSPAIKKGILQTVKVVDELVKVMGRHKPENIVIEMAR

ENQTTQKGQKNSRERMKRIEEGIKELGSQILKEHPVENTQLQNEKLYLYYLQNGRDMYVDQELDINRLS

DYDVDHIVPQSFLKDDSIDNKVLTRSDKNRGKSDNVPSEEVVKKMKNYWRQLLNAKLITQRKFDNLTKA

ERGGLSELDKAGFIKRQLVETRQITKHVAQILDSRMNTKYDENDKLIREVKVITLKSKLVSDFRKDFQF

YKVREINNYHHAHDAYLNAVVGTALIKKYPKLESEFVYGDYKVYDVRKMIAKSEQEIGKATAKYFFYSN

IMNFFKTEITLANGEIRKRPLIETNGETGEIVWDKGRDFATVRKVLSMPQVNIVKKTEVQTGGFSKESI

LPKRNSDKLIARKKDWDPKKYGGFDSPTVAYSVLVVAKVEKGKSKKLKSVKELLGITIMERSSFEKNPI

DFLEAKGYKEVKKDLIIKLPKYSLFELENGRKRMLASAGELQKGNELALPSKYVNFLYLASHYEKLKGS

PEDNEQKQLFVEQHKHYLDEIIEQISEFSKRVILADANLDKVLSAYNKHRDKPIREQAENIIHLFTLTN
LGAPAAFKYFDTTIDRKRYTSTKEVLDATLIHQSITGLYETRIDLSQLGGDSGGSTNLSDIIEKETGKQ
LVIQESILMLPEEVEEVIGNKPESDILVHTAYDESTDENVMLLTSDAPEYKPWALVIQDSNGENKIKML
SGGSPKKKRKV*

pCMV-dSaCas9 (dSaCas9-linker-UGI-NLS)

KRNYILGLDIGITSVGYGIIDYETRDVIDAGVRLFKEANVENNEGRRSKRGARRLKRRRRHRIQRVKKL
LFDYNLLTDHSELSGINPYEARVKGLSQKLSEEEFSAALLHLAKRRGVHNVNEVEEDTGNELSTKEQIS
RNSKALEEKYVAELQLERLKKDGEVRGSINRFKTSDYVKEAKQLLKVQKAYHQLDQSFIDTYIDLLETR
RTYYEGPGEGSPFGWKDIKEWYEMLMGHCTYFPEELRSVKYAYNADLYNALNDLNNLVITRDENEKLEY
YEKFQIIENVFKQKKKPTLKQIAKEILVNEEDIKGYRVTSTGKPEFTNLKVYHDIKDITARKEIIENAE
LLDQIAKILTIYQSSEDIQEELTNLNSELTQEEIEQISNLKGYTGTHNLSLKAINLILDELWHTNDNQI
AIFNRLKLVPKKVDLSQQKEIPTTLVDDFILSPVVKRSFIQSIKVINAIIKKYGLPNDIIIELAREKNS
KDAQKMINEMQKRNRQTNERIEEIIRTTGKENAKYLIEKIKLHDMQEGKCLYSLEAIPLEDLLNNPFNY
EVDHIIPRSVSFDNSFNNKVLVKQEENSKKGNRTPFQYLSSSDSKISYETFKKHILNLAKGKGRISKTK
KEYLLEERDINRFSVQKDFINRNLVDTRYATRGLMNLLRSYFRVNNLDVKVKSINGGFTSFLRRKWKFK
KERNKGYKHHAEDALIIANADFIFKEWKKLDKAKKVMENQMFEEKQAESMPEIETEQEYKEIFITPHQI
KHIKDFKDYKYSHRVDKKPNRELINDTLYSTRKDDKGNTLIVNNLNGLYDKDNDKLKKLINKSPEKLLM
YHHDPQTYQKLKLIMEQYGDEKNPLYKYYEETGNYLTKYSKKDNGPVIKKIKYYGNKLNAHLDITDDYP
NSRNKVVKLSLKPYRFDVYLDNGVYKFVTVKNLDVIKKENYYEVNSKCYEEAKKLKKISNQAEFIASFY
NNDLIKINGELYRVIGVNNDLLNRIEVNMIDITYREYLENMNDKRPPRIIKTIASKTQSIKKYSTDILG
NLYEVKSKKHPQIIKKGSGGSTNLSDIIEKETGKQLVIQESILMLPEEVEEVIGNKPESDILVHTAYDE
STDENVMLLTSDAPEYKPWALVIQDSNGENKIKMLSGGSPKKKRKV*

pCMV-AdeI (AdeI-linker-NLS)

MATTSHVEHELLSKLIDELKVKANSDPEADVLAGRLLHRLKAESVTHTVAEYLEVFSDKFYDEEFFQMH
RDELETRVSAFAQSPAYERIVSSGYLSALRYYDTYLYVGRSGKQESVQHFYMRLAGFCASTTCLYAGLR
AALQRARPEIESDMEVFDYYFEHLTSQTVCCSTPFMRFAGVENSTLASCILTTPDLSSEWDVTQALYRH
LGRYLFQRAGVGVGVTGAGQDGKHISLLMRMINSHVEYHNYGCKRPVSVAAYMEPWHSQIFKFLETKLP
ENHERCPGIFTGLFVPELFFKLFRDTPWSDWYLFDPKDAGDLERLYGEEFEREYYRLVTAGKFCGRVSI
KSLMFSIVNCAVKAGSPFILLKEACNAHFWRDLQGEAMNAANLCAEVLQPSRKSVATCNLANICLPRCL
VNAPLAVRAQRADTQGDELLLALPRLSVTLPGEGAVGDGFSLARLRDATQCATFVVACSILQGSPTYDS
RDMASMGLGVQGLADVFADLGWQYTDPPSRSLNKEIFEHMYFTALCTSSLIGLHTRKIFPGFKQSKYAG
GWFHWHDWAGTDLSIPREIWSRLSERIVRDGLFNSQFIALMPTSGCAQVTGCSDAFYPFYANASTKVTN
KEEALRPNRSFWRHVRLDDREALNLVGGRVSCLPEALRQRYLRFQTAFDYNQEDLIQMSRDRAPFVDQS
QSHSLFLREEDAARASTLANLLVRSYELGLKTIMYYCRIEKAADLGVMECKASAALSVPREEQNERSPA

EQMPPRPMEPAQVAGPVDIMSKGPGEGPGGWCVPGGLEVCYKYRQLFSEDDLLETDGFTERACESCQSG
GSSGGSPKKKRKV*
